# Culture-based diversity of endophytic fungi of three species of *Ferula* grown in Iran

**DOI:** 10.3389/fmicb.2024.1363158

**Published:** 2024-05-23

**Authors:** Naser Safaie, Mina Salehi, Mona Felegari, Siamak Farhadi, Samira Karimzadeh, Sadegh Asadi, Jun-Li Yang, Mohammad Reza Naghavi

**Affiliations:** ^1^Department of Plant Pathology, Faculty of Agriculture, Tarbiat Modares University, Tehran, Iran; ^2^Department of Plant Genetics and Breeding, Faculty of Agriculture, Tarbiat Modares University, Tehran, Iran; ^3^Seed and Plant Improvement Institute, Agricultural Research, Education and Extension Organization (AREEO), Karaj, Iran; ^4^Division of Crop Ecology, Department of Agronomy and Plant Breeding, Faculty of Agriculture & Natural Resources, University of Tehran, Karaj, Iran; ^5^CAS Key Laboratory of Chemistry of Northwestern Plant Resources and Key Laboratory for Natural Medicine of Gansu Province, Lanzhou Institute of Chemical Physics, Chinese Academy of Sciences, Lanzhou, China; ^6^Division of Biotechnology, Department of Agronomy and Plant Breeding, College of Agriculture and Natural Resources, University of Tehran, Karaj, Iran

**Keywords:** dominance and evenness indices, mycoflora, *Ferula ovina*, *F. galbaniflua*, *F. persica*, species richness

## Abstract

A total of 1,348 endophytic fungal strains were isolated from *Ferula ovina*, *F. galbaniflua*, and *F. persica*. They included *Eurotiales* (16 species), *Pleosporales* (11 species), *Botryosphaeriales* (1 species), *Cladosporiales* (2 species), *Helotiales* (6 species), *Hypocreales* (31 species), *Sordariales* (7 species), *Glomerellales* (2 species), and *Polyporales* (1 species). *F. ovina* had the richest species composition of endophytic fungi, and the endophytic fungi were most abundant in their roots compared to shoots. Chao, Margalef, Shannon, Simpson, Berger–Parker, Menhinick, and Camargo indices showed that *F. ovina* roots had the most endophytic fungal species. The frequency distribution of fungal species isolated from *Ferula* spp. fell into the log-series model, and *F. ovina* roots had the highest Fisher alpha. The dominance indices showed that there are no dominant species in the endophytic fungal community isolated from *Ferula* spp., indicating community stability. Evenness values were 0.69, 0.90, 0.94, and 0.57 for endophytic fungi isolated from *F. ovina* roots, *F. ovina* shoots, *F. galbaniflua* roots, and *F. persica* roots, respectively, indicating a species distribution that tends toward evenness. The fungal species community isolated from each of *F. ovina* roots, *F. ovina* shoots, *F. galbaniflua* roots, and *F. persica* roots was a diverse species group originating from a homogeneous habitat. Their distribution followed a log-normal distribution, suggesting that the interactions of numerous independent environmental factors multiplicatively control species abundances. Principal component analysis showed that the highest species diversity and dominance were observed in the endophytic fungal community isolated from *F. ovina* and *F. persica* roots, respectively.

## 1 Introduction

Plants are not solitary beings but consist of an incredible diversity of microorganisms living nearby, particularly those that live inside them and have coevolved together during billions of years of coexistence. Microorganisms known as endophytes live asymptomatically for at least a period of their life cycles inside plants (Hardoim et al., 2015). Endophytic fungi are frequent and diversified polyphyletic microorganisms inhabiting plant tissues (Faeth and Fagan, 2002). Diverse fungal endophytes living within plants can enhance host fitness, the production of secondary metabolites (Jia et al., 2016; Kaul et al., 2016; Salehi et al., 2021; Salehi and Farhadi, 2022), stress tolerance (Rodriguez et al., 2008), disease resistance (Busby et al., 2016; Nourian et al., 2024; Ranjbar et al., 2024), and biomass accumulation (Mucciarelli et al., 2003). The great diversity of fungal endophytes implies their highly intricate and different functional characteristics (Torres-Cortes et al., 2018). The studies displayed that endophytic fungi communities diversified depending on plant tissues (Wearn et al., 2012) and host genotypes (Matsumura and Fukuda, 2013). There are an estimated 300,000 species of higher plants on earth, and each one harbors one or more obligatory endophytic fungi, some of which colonize particular hosts (Nisa et al., 2015). Only a small number of plant species have been investigated in terms of endophytic fungal communities. Endophytes may be hyperdiverse because it has been suggested that there could be up to a million different endophytic fungal taxa (Nisa et al., 2015). There are only a handful of described populations of endophytic fungi. Indeed, exploring the endophytic fungi is a long way ahead.

*Ferula*, one of the largest and most popular genera of Apiaceae, has numerous species frequently exploited in traditional medicine and is a prospective source of biologically active compounds including anti-inflammatory, antiviral, antidiabetic, antitumor, cytotoxic, anticancer, estrogenic, anti-ulcerative, antibacterial, acaricidal, antiglycation, and antiprotozoal (Mohammadhosseini et al., 2019; Salehi et al., 2019c; Sonigra and Meena, 2021). *Ferula* is widely distributed throughout the Mediterranean, the Far East, north India, and southwest and central Asia (particularly Iran and Afghanistan; Sonigra and Meena, 2021). More than 30 *Ferula* spp. have been identified in Iran, with approximately half of them endemic and/or native, including *F. ovina*, *F. persica*, and *F. galbaniflua* (synonyms of *F. gummosa*; Safaian and Shokri, 1993; Panahi and Mahmoodi, 2021). Umbelliprenin, auraptene, galbanic acid, ferutinin, daucane esters, ferulenol, sinkiangenorin C, sinkiangenorin E, farnesiferol A, farnesiferol B, and ferprenin are some of the hopeful bioactive compounds found in *Ferula* spp.; consequently, *Ferula* species are considered valuable sources for the development of plant-derived medicines, particularly chemopreventive ones (Salehi et al., 2019c).

In light of the wide range of medicinal properties of *Ferula* spp. and the fact that the exploration of the endophytic fungi is a long way off, and likewise, the endophytic fungi of *F. ovina*, *F. persica*, and *F. galbaniflua* have not been comprehensively surveyed, studying their endophytic fungal diversity is essential.

The aims of this study were (a) to isolate endophytic fungi from *F. ovina*, *F. persica*, and *F. galbaniflua* grown in Iran, (b) to morphologically and molecularly identify the endophytic fungi isolated from roots and shoots of *F. ovina*, *F. persica*, and *F. galbaniflua*, and (c) to investigate the possible effect of different tissues (roots and shoots) and *Ferula* species on endophytic fungi communities.

## 2 Materials and methods

### 2.1 Sampling and fungal isolation

Healthy samples, including the roots and shoots, were collected from *F. ovina*, *F. persica*, and *F. galbaniflua* grown in their major habitats in Iran in June 2022. Asymptomatic roots and shoots were cut by mentioning *Ferula* species, sample code, and taken in paper bags to the lab. The samples were treated for 1 min with 75% ethanol (v/v) and 2 min with 2.5% sodium hypochlorite (w/v), followed by two rinses with sterile water. To evaluate the surface sterilization effectiveness, the final rinse water (10 mL) was centrifuged at 10,000*g* for 10 min. The supernatant was taken off and streaked onto potato dextrose agar (PDA) with 250 mg/L of chloramphenicol (CAM) (PDACAM). No mycelial development took place, which validated the surface sterilization. Small (4 mm^2^) surface-sterilized sections of root and shoot segments were cut and placed on the PDACAM surface in special Petri dishes (100 × 15 mm), then incubated at 25°C to promote the growth of endophytic fungi. The single spore or hyphal tip culture (Tutte, 1969) was used to obtain pure cultures of the fungal isolates. Fo#, Fp#, and Fg# series were used to number all fungal endophytes isolated from *F. ovina, F. persica, and F. galbaniflua*, respectively. They were stored on PDA at 4°C.

### 2.2 Morphological identification of endophytic fungi

The macroscopic examination of colony characteristics, by observing topography, color, and texture, as well as color and topography of colony back, and the microscopic investigation of conidia, conidiophores, and spore arrangement, using lactophenol and lactophenol cotton blue staining, under a compound microscope, were both used to study fungal morphology (Aneja, 2007; Dos Reis et al., 2022). Photomicrographs were taken with an Olympus DP72 digital camera attached to an Olympus BX51 microscope equipped with differential interference contrast.

### 2.3 Molecular identification of endophytic fungi

Endophytic fungi were grown in potato dextrose broth (PDB) at 25°C with continuous shaking. Genomic DNA extraction was performed from freeze-dried mycelia as described previously (Salehi et al., 2018, 2019a,b, 2020a,b; Farhadi et al., 2020a,b; Nourian et al., 2021; Jahedi et al., 2023). The fungal internal transcribed spacer (ITS) region (ITS1-5.8S-ITS2) was amplified, using polymerase chain reaction (PCR), by the universal primers ITS1 and ITS4. One microliter of genomic DNA (~10 ng), 1 μL of forward and reverse primers (10 pM), 12.5 μL of Premix Taq (TaKaRa Biotechnology Ltd., Japan), and 10.5 μL of PCR-grade water were all included in the PCR reaction mixtures (25 μL). The PCR reaction programs were as follows: initial denaturation at 94°C for 3 min, 30 cycles of denaturation (94°C for 30 s), annealing (56°C for 30 s), extension (72°C for 1 min), and final extension (72°C for 5 min). PCR reaction success was assessed through agarose gel electrophoresis, and then the PCR products were purified using a DNA gel extraction kit (Axygen Biotechnology Ltd., China). Direct sequencing of purified PCR products was conducted by the same primers using Bioneer (Shanghai, China).

### 2.4 Diversity analyses of cultivable endophytic fungi

Two key factors—evenness and richness—should be considered when determining how diverse a sample is (Kim et al., 2017). Diversity typically increases along with increases in species evenness and richness. The diversity of endophytic fungi in the different tissues of *F. ovina*, *F. persica*, and *F. galbaniflua* was examined at the species level. The endophytic fungal composition and abundance of endophytic fungi from each tissue of *Ferula* spp. must be assessed using the number of endophytic fungal isolates (*N*) and species as the statistical unit. The macroscopic examination enables the classification of morphotypes.

Here, abundance (*N*) is the number of isolates, whereas richness (*S*) signifies the number of species in the endophytic fungal community of a specific sample type (roots and shoots from *F. ovina*, *F. persica*, and *F. galbaniflua*).

To assess the propriety of each tissue type of each *Ferula* species as a substrate for fungal colonization, colonized samples (i.e., colonized by one endophytic fungus, at minimum) were enumerated and colonization frequency (CF; also known as isolation rate) was calculated according to Eq. 1 (Hata and Futai, 1995). CF was estimated as the number of sections colonized by endophytic fungi divided by the total number of incubated sections (Eq. 1, Huang et al., 2008).

(1) $$CF\;\left(\%\right)=\frac{\mathrm{No}.\;\mathrm{of}\;\mathrm{colonized}\;\mathrm{samples}}{\mathrm{No}.\;\mathrm{of}\;\mathrm{plated}\;\mathrm{samples}}\times 100$$

Isolation frequency (IF), the frequency of the occurrence of particular endophytic fungal species in total isolates, is relative abundance (RA%) computed as the number of whole isolates of a particular species divided by the sum of all isolates across all species (Eq. 2; Huang et al., 2008).

(2) $$RA\;\left(\%\right)=\frac{\mathrm{No}.\;\mathrm{of}\;\mathrm{isolates}\;\mathrm{of}\;a\;\mathrm{particular}\;\mathrm{species}}{\;\mathrm{Sum}\;\mathrm{of}\;\mathrm{isolates}\;\mathrm{of}\;\mathrm{all}\;\mathrm{species}}\times 100$$

A non-parametric method to approximate the species number in a community is Chao1. The foundation of the Chao richness estimator, created by Anne Chao, is the idea that rare species can reveal the greatest information regarding missing species numbers. Since the Chao richness estimator favors the low-abundance species, merely the singleton(s) and doubleton(s) are applied to approximate the missing species number. Consequently, data sets that are biased toward low-abundance species benefit most from this index (Kim et al., 2017).

The Chao richness estimator is estimated by Eq. 3 (Chao, 1984).

(3) $$S_{\mathrm{chao}1}=S_{obs}+\frac{F_{1}\;\left(F_{1}-1\right)\;}{\;2\;\left(F_{2}+1\right)}$$

Where *F*_1_ and *F*_2_ denote the number of singleton(s) and doubleton(s), respectively, and *S_obs_* signifies the observed species number.

The Menhinick index (*D_mn_*), which measures species richness among the endophytic fungal isolates, is calculated using Eq. 4 (Whittaker, 1977).

(4) $$D_{mn}=\frac{S\;}{\;\sqrt{N}}$$

Where *S* signifies the number of different endophytic fungal species in a given sample and *N* is the total number of endophytic fungal isolates in a given sample.

The Camargo evenness index (Eq. 5) was calculated to determine fungal dominance.

(5) $$EC=\frac{1\;}{\;D_{mn}}$$

Where *D_mn_* is species richness.

A species is considered dominant if its RA is greater than the Camargo evenness index (*p_i_* >1/ *D_mn_*; Camargo, 1992).

The Shannon diversity index was developed to examine the diversity of the fungal endophyte communities in each tissue (Eq. 6; Shannon, 1948):

(6) $$H^{\prime }=-\overset{s}{\underset{i=1}{\sum }}p_{i}\ln p_{i}$$

Where *p_i_* denotes the RA of a species in a given tissue. *H*′ could range from 0 (there is only one species, and each individual will be of that species, without a doubt) to higher values, indicating high uncertainty because species are distributed fairly evenly.

Pielou’s evenness index was calculated as follows (Eq. 7).

(7) $$J=\frac{H^{\prime }}{\ln\;\left(S\right)}$$

Where *J* is Pielou’s measure of species evenness, *H*′ signifies the Shannon–Wiener index in the given sample, and *S* denotes the total number of species in the given sample.

The evenness index (*E*) was calculated using Eq. 8 (Muthukrishnan et al., 2012).

(8) $$E=\frac{e^{H\prime }}{S}$$

Sorenson index of similarity, the proportion of twice the number of the species shared between two communities to the sum of the number of species in each community, was calculated using Eq. 9 (Sorensen, 1948). Where *a* signifies the number of common species of endophytic fungi in two samples, and *b* and *c* denote the species specifically found in each of the samples.

(9) $$\beta_{Sor}=\frac{2a}{2a+b+c}$$

Jaccard similarity index, the ratio of species shared between two communities to the total number of species in all, was estimated by Eq. 10 (Jaccard, 1912), where *a* denotes the number of common endophytic fungal species in two samples and *b* and *c* are the number of species specifically found in each of the samples.

(10) $$\beta_{Jac}=\frac{c}{\left(a+b+c\right)}$$

A parametric index called Fisher alpha supposes that species abundances have a log-series distribution. In Eq. 11, *S* denotes the number of species, *n* denotes the number of endophytic fungal isolates, and *α* denotes Fisher alpha (Fisher et al., 1943). Furthermore, species frequency/abundance distribution for endophytic fungal strains isolated from *F. ovina*, *F. galbaniflua,* and *F. persica* was calculated according to a logarithmic series evolved by Fisher et al. (1943).

(11) $$S=\alpha \times \ln\left(1+\frac{n}{\alpha }\right)$$

Berger–Parker dominance index (*d*; Eq. 12) displays the numerical importance of dominant species (most abundant species).

(12) $$d=\frac{n_{\text{max}}}{N}$$

Where *n_max_* denotes the number of individuals in the dominant species, and *N* denotes the total number of individuals in the sample.

Reciprocal of Berger–Parker (1/*d*) is frequently used, and its increment implies diversity increment and dominance decrement.

Furthermore, the Simpson dominance index (Eq. 13; Simpson, 1949) was used for evaluating species diversity. It shows the likelihood that two isolates randomly chosen from a sample will be of the same species.

(13) $$D=\frac{\sum_{1}^{s}n_{i}\;\left(n_{i}-1\right)\;}{N\;\left(N-1\right)}$$

Where *n_i_* is the total number of isolates falling into the species *i*. *N* signifies the total number of isolates of all species. Moreover, the complementarity of the Simpson diversity index (1-*D*) and the reciprocal of the Simpson index (1/*D*) were calculated.

Probability of interspecific encounter (PIE; Eq. 14) is a metric introduced by Hurlbert (1971) and basically asks, “what is the probability that two individuals randomly selected from a community are of different species?” A community with more even species RAs displays a higher PIE than that with dominant species. Indeed, a low PIE shows that a few species dominate and a high PIE displays community evenness.

(14) $$\mathrm{PIE}=\left(\frac{N}{N-1}\right)\left(1-\overset{S}{\underset{1}{\sum }}P_{i}^{2}\right)$$

Where *N* is the total number of individuals, and *P_i_* signifies the proportion of species *i* in the sample.

Species richness was measured by the Margalef index (Eq. 15; Margalef, 1958).

(15) $$D_{\mathrm{Mg}}=\frac{S-1}{\ln\left(N\right)}$$

Where *S* represents the number of species, and *N* is the total number of isolates.

The data were analyzed using Excel (2013) and Minitab (18.1). GraphPad Prism (GraphPad Prism 5), Excel (Excel, 2013), and PowerPoint (2013) software were used for making graphs.

## 3 Results

### 3.1 Isolation rate and classification of endophytic fungi

No fungal colonies were observed on PDACAM plates in the control group (surface sterilization validation), which ascertained the surface of *F. ovina*, *F. galbaniflua,* and *F. persica* tissues were completely disinfected, and consequently, the fungi isolated in the present study were all endophyte. A total of 1,348 endophytic fungal strains, which belonged to 2 phyla, 5 classes, 9 orders, 18 families, and 24 genera (Supplementary Figure S1), were isolated from 3,240 cultured tissue segments of *F. ovina*, *F. galbaniflua,* and *F. persica* tissues growing naturally in Iran (Supplementary Table S1). The endophytic fungal isolates were investigated morphologically, and 77 representative isolates of different morphotypes (Supplementary Figures S2, S3 and Supplementary Table S2) were considered for detailed description and morphological and molecular identification. The RA% of endophytic fungi isolated from the different species of *Ferula* (*F. ovina*, *F. galbaniflua,* and *F. persica*) is presented in Supplementary Figure S4. One thousand twenty-seven endophytic fungal strains were isolated from *F. ovina*, with a CF of 22.96% (142 strains from shoots, 12.59%; 885 strains from roots, 33.33%; Supplementary Table S1). One hundred eighty-seven endophytic fungal strains were isolated from *F. galbaniflua* roots, and CF was 32.78% (Supplementary Table S1). Accordingly, 134 strains were isolated from *F. persica*, and the CF was 32.22% (Supplementary Table S1). *F. ovina* had the highest CF (22.96%) and the most isolated endophytic fungal strains (1027), followed by *F. galbaniflua* (16.39%) and *F. persica* (16.11%; Supplementary Table S1). In terms of tissue, the most endophytic fungal strains were isolated from the roots of *F. ovina*, *F. galbaniflua*, and *F. persica* (1206). No endophytic fungal strain was isolated from seeds of the three mentioned species of *Ferula* as well as shoots of *F. galbaniflua* and *F. persica* (Supplementary Table S1).

**Figure 1:**
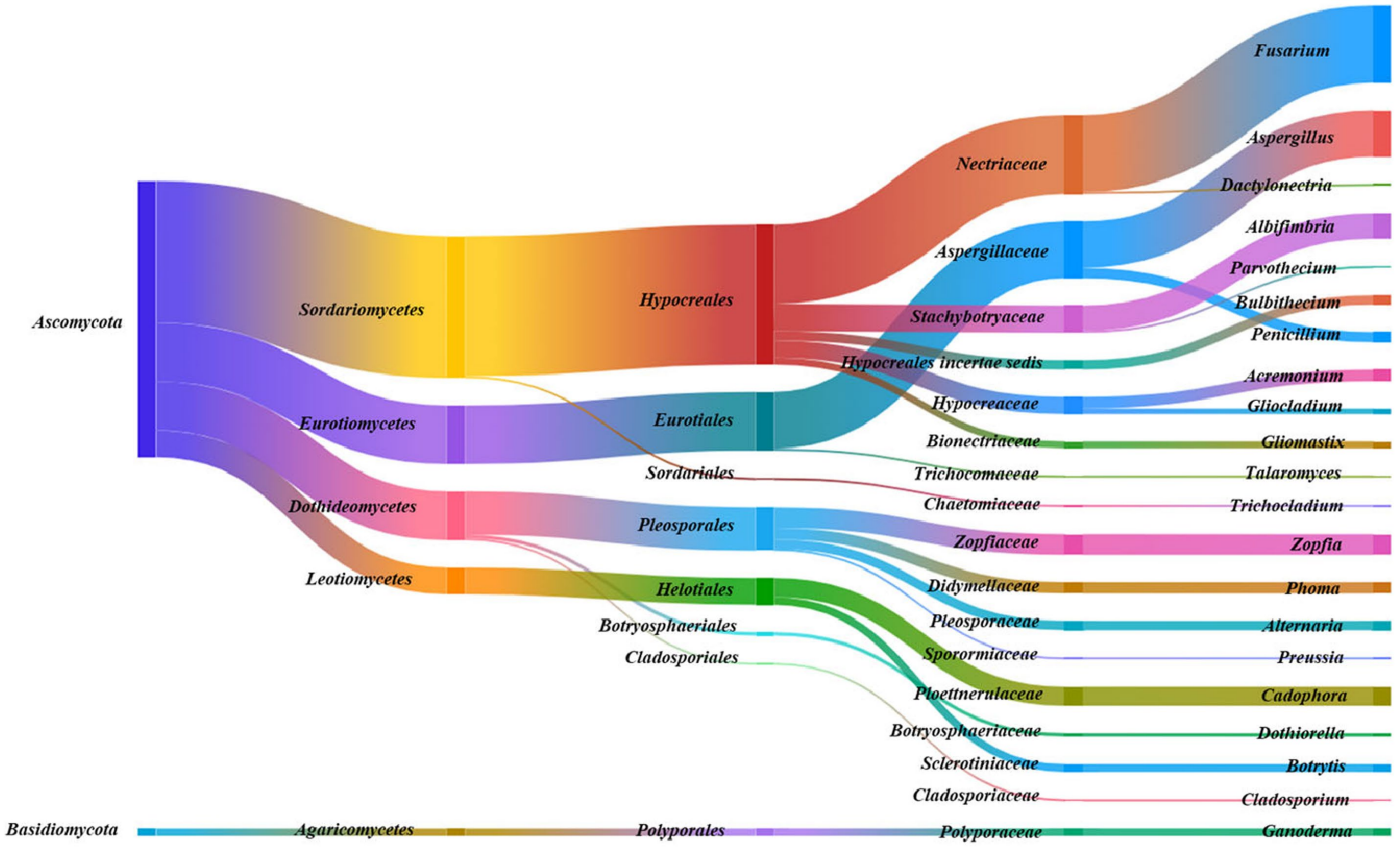
Species taxonomic relationship. The endophytic fungi isolated from *Ferula ovina* shoots and roots belonged to 2 phyla, 5 classes, 8 orders, 17 families, and 21 genera.

**Figure 2:**
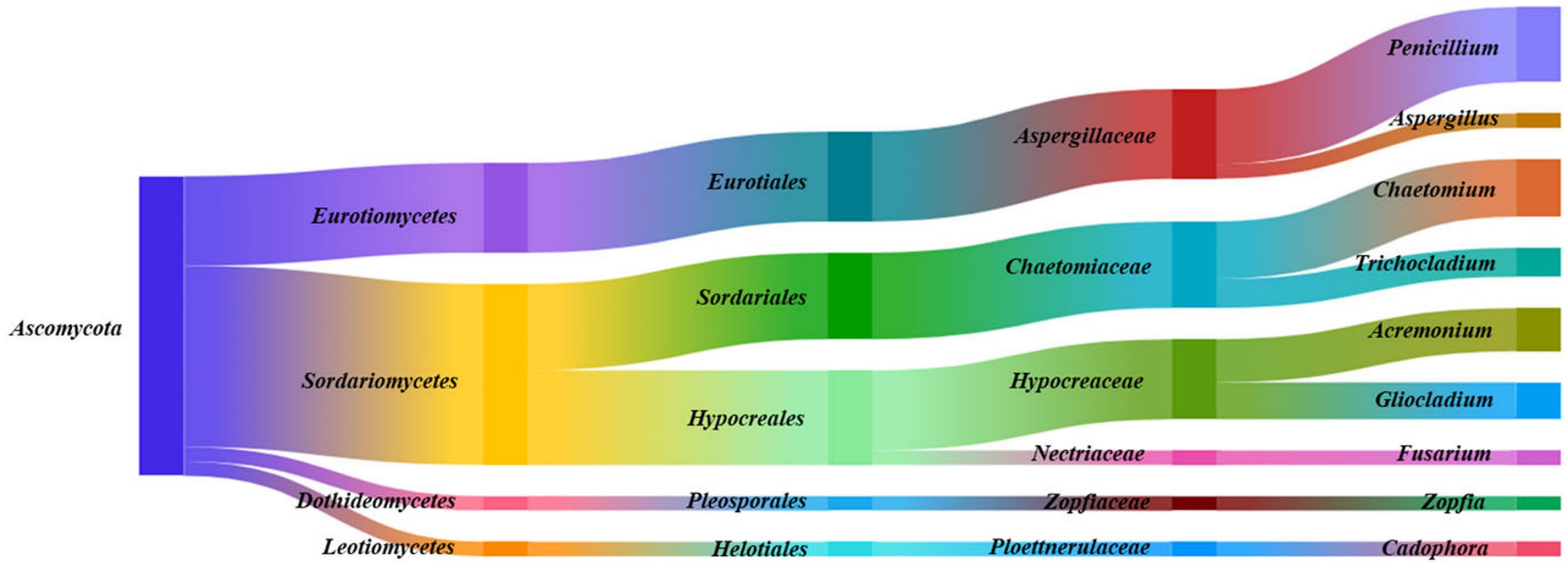
Species taxonomic relationship. The endophytic fungi isolated from *Ferula galbaniflua* roots belonged to one phylum, four classes, five orders, six families, and nine genera.

**Figure 3:**
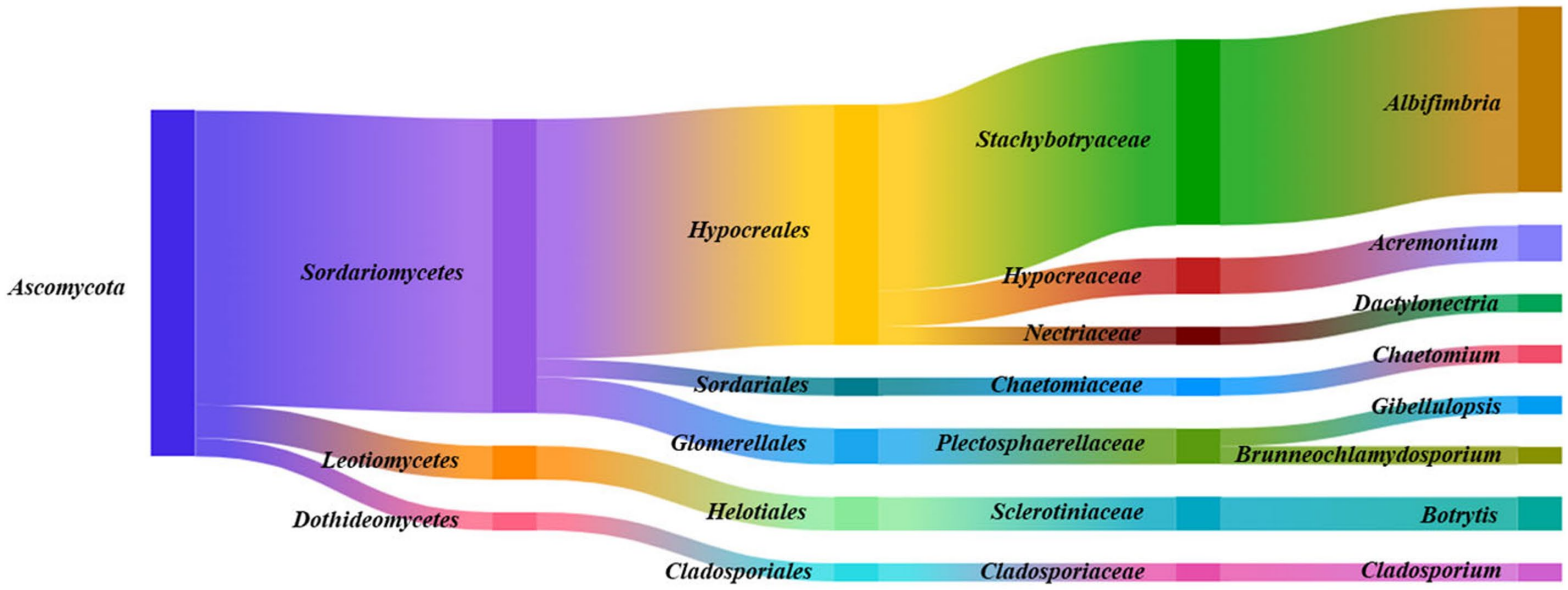
Species taxonomic relationship. The endophytic fungi isolated from *Ferula persica* roots belonged to one phylum, three classes, five orders, seven families, and eight genera.

As shown in Figures 1–3, the culturable endophytic fungi in *F. ovina* displayed rich diversity compared with *F. galbaniflua* and *F. persica*. The morphology and molecular identification showed that 1,027 endophytic fungi isolated from *F. ovina* belonged to 2 Phyla (*Ascomycota* and *Basidiomycota*), 5 classes, 8 orders, 17 families, and 21 genera. There were 28 (2.73%) endophytic fungal strains in the phylum of *Basidiomycota* belonging to the class of *Agaricomycetes*, order of *Polyporales,* family of *Polyporaceae*, and genus of *Ganoderma*. *Sordariomycetes* (513 isolates, 49.95%) was the dominant class in *Ascomycota*, followed by *Eurotiomycetes* (212 isolates, 20.64%), *Dothideomycetes* (177 isolates, 17.23%), and *Leotiomycetes* (97 isolates, 9.44%). *Pezizomycotina* (999 isolates) was the only subphylum in the phylum of *Ascomycota*. The relative frequency of *Pezizomycotina* was 97.27%, including *Eurotiales* (212 isolates, 20.64%), *Pleosporales* (157 isolates, 15.29%), *Botryosphaeriales* (13 strains, 1.27%), *Cladosporiales* (7 isolates, 0.68%), *Helotiales* (97 isolates, 9.44%), *Hypocreales* (506 isolates, 49.27%), and *Sordariales* (7 isolates, 0.68%). Nectriaceae (286 isolates, 27.85%) and *Aspergillaceae* (205 isolates, 19.96%) were the dominant families. *Fusarium* (279 isolates, 27.17%) and *Aspergillus* (168 isolates, 16.36%) were the dominant genera (Figure 1).

Endophytic fungal strains (187) isolated from *F. galbaniflua* roots were classified into one phylum (*Ascomycota*), four classes (*Sordariomycetes*, *Eurotiomycetes*, *Dothideomycetes*, and *Leotiomycetes*), five orders (*Hypocreales*, *Eurotiales*, *Sordariales*, *Pleosporales*, and *Helotiales*), six families (*Aspergillaceae*, *Chaetomiaceae*, *Hypocreaceae*, *Zopfiaceae*, *Ploettnerulaceae*, and *Nectriaceae*), and nine genera (*Penicillium*, *Chaetomium*, *Acremonium*, *Gliocladium*, *Trichocladium*, *Aspergillus*, *Zopfia*, *Cadophora*, and *Fusarium*). *Sordariomycetes* (113 isolates, 60.43%), *Hypocreales* (59 isolates, 31.55%), *Aspergillaceae* (56 isolates, 29.95%), and *Penicillium* sp. (47 isolates, 25.13%) were the dominant phylum, class, order, family, and genus, respectively (Figure 2).

A total of 134 endophytic fungal strains were isolated from *F. persica* and classified into one phylum (*Ascomycota*), three classes (*Sordariomycetes*, *Leotiomycetes*, and *Dothideomycetes*), five orders (*Hypocreales*, *Glomerellales*, *Helotiales*, *Sordariales*, and *Cladosporiale*s), seven families (*Stachybotryaceae*, *Hypocreaceae*, *Plectosphaerellaceae*, *Sclerotiniaceae*, *Nectriaceae*, *Chaetomiaceae*, and *Cladosporiaceae*), and eight genera (*Albifimbria*, *Acremonium*, *Botrytis*, *Dactylonectria*, *Chaetomium*, *Gibellulopsis*, *Brunneochlamydosporium*, and *Cladosporium*; Figure 3). As shown in Figure 3, *Sordariomycetes*, *Hypocreales*, *Stachybotryaceae*, and *Albifimbria* were the dominant class, order, family, and genus, respectively.

**Figure 4:**
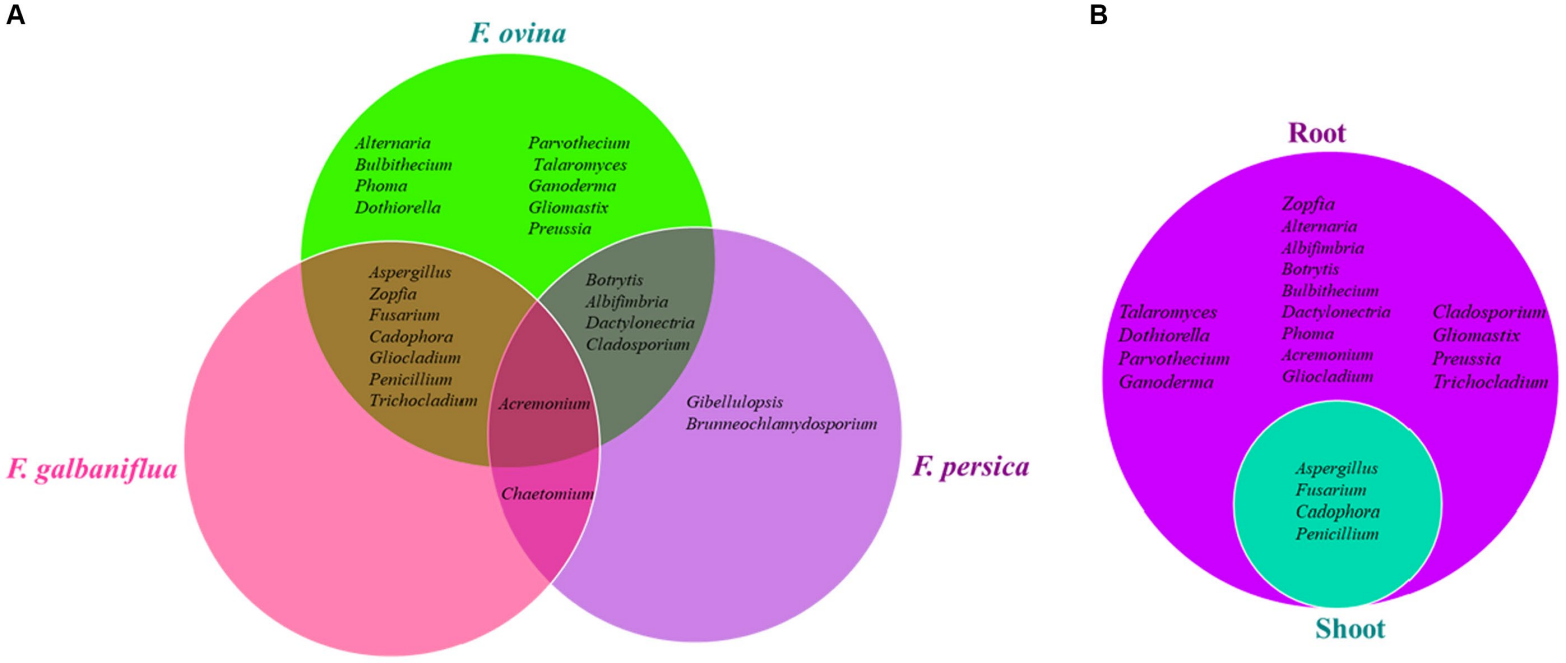
Venn diagram of endemic and common endophytic fungal genera isolated from *Ferula ovina*, *F. galbaniflua*, *F. persica* **(A)**, and *F. ovina* shoots and roots **(B)**.

The genus *Acremonium* was isolated from all *Ferula* species (*F. ovina*, *F. galbaniflua,* and *F. persica*; Figure 4A), accounting for an RA of 4.09, 14.44, and 10.45%, respectively (Supplementary Figures S5–S7). *Alternaria*, *Bulbithecium*, *Phoma*, *Dothiorella*, *Parvothecium*, *Talaromyces*, *Ganoderma*, *Gliomastix*, and *Preussia* were only isolated from *F. ovina* (Figure 4A). Furthermore, *Gibellulopsis* and *Brunneochlamydosporium* were endemic to *F. persica* (Figure 4A). Additionally, *Aspergillus*, *Zopfia*, *Fusarium*, *Cadophora*, *Gliocladium*, *Penicillium*, and *Trichocladium* were only found in *F. ovina* and *F. galbaniflua* (Figure 4A). Besides, *Botrytis*, *Albifimbria*, *Dactylonectria*, and *Cladosporium* were only present in *F. ovina* and *F. persica* (Figure 4A). Furthermore, *Chaetomium* is shared between *F. galbaniflua* and *F. persica* roots (Figure 4A). Of 24 genera, 21, 9, and 8 fungal genera were isolated from *F. ovina*, *F. galbaniflua*, and *F. persica*, respectively (Figure 4A).

As shown in Supplementary Figure S4, 885 (86.17) and 142 (13.83) endophytic fungal strains were isolated from *F. ovina* roots and shoots, respectively. Among them, the roots and shoots of *F. ovina* shared only four genera: *Aspergillus*, *Fusarium*, *Cadophora*, and *Penicillium* (Figure 4B). *Talaromyces*, *Dothiorella*, *Parvothecium*, *Ganoderma*, *Zopfia*, *Alternaria*, *Albifimbria*, *Botrytis*, *Bulbithecium*, *Dactylonectria*, *Phoma*, *Acremonium*, *Gliocladium*, *Cladosporium*, *Gliomastix*, *Preussia*, and *Trichocladium* were endemic to *F. ovina* roots but not isolated from its shoots (Figure 4B). *Fusarium* was a dominant and common genus isolated from the root and shoot tissues of *F. ovina* with an RF of 24.75 and 42.25%, respectively (Supplementary Figure S5). Except for *Fusarium*, the dominant endophytic fungal genus of *F. ovina* was *Aspergillus,* showing an RF of 12.43 and 40.85% in roots and shoots, respectively (Supplementary Figure S5). Depending on the type of tissue, the endophytic fungal communities varied greatly (Supplementary Figure S5). No fungal endophytes were isolated from the shoots of *F. galbaniflua* and *F. persica*.

The fungal endophytes of *F. ovina* roots were assigned the most abundant of 21 genera.

### 3.2 Alpha diversity of endophytic fungi isolated from *F. ovina*, *F. galbaniflua*, and *F. persica*

Fungal species isolated from *F. ovina*, *F. galbaniflua,* and *F. persica* are presented in Supplementary Figure S8. Shannon index, *H*, is a diversity one considering both the total number of endophytic fungi and the fungal species number. For communities with a single species, it is equal to 0, while for communities with multiple taxa, each with a small number of members, it is higher. Shannon–Wiener index and Simpson dominance index displayed that the endophytic fungal community isolated from *F. ovina* roots had the highest diversity (*H* = 3.67 and *D* = 0.036) and *F. persica* the lowest (*H* = 1.64 and *D* = 0.317). The results implied that the species diversity of culturable endophytic fungi in *Ferula* was affected by the host.

**Figure 5:**
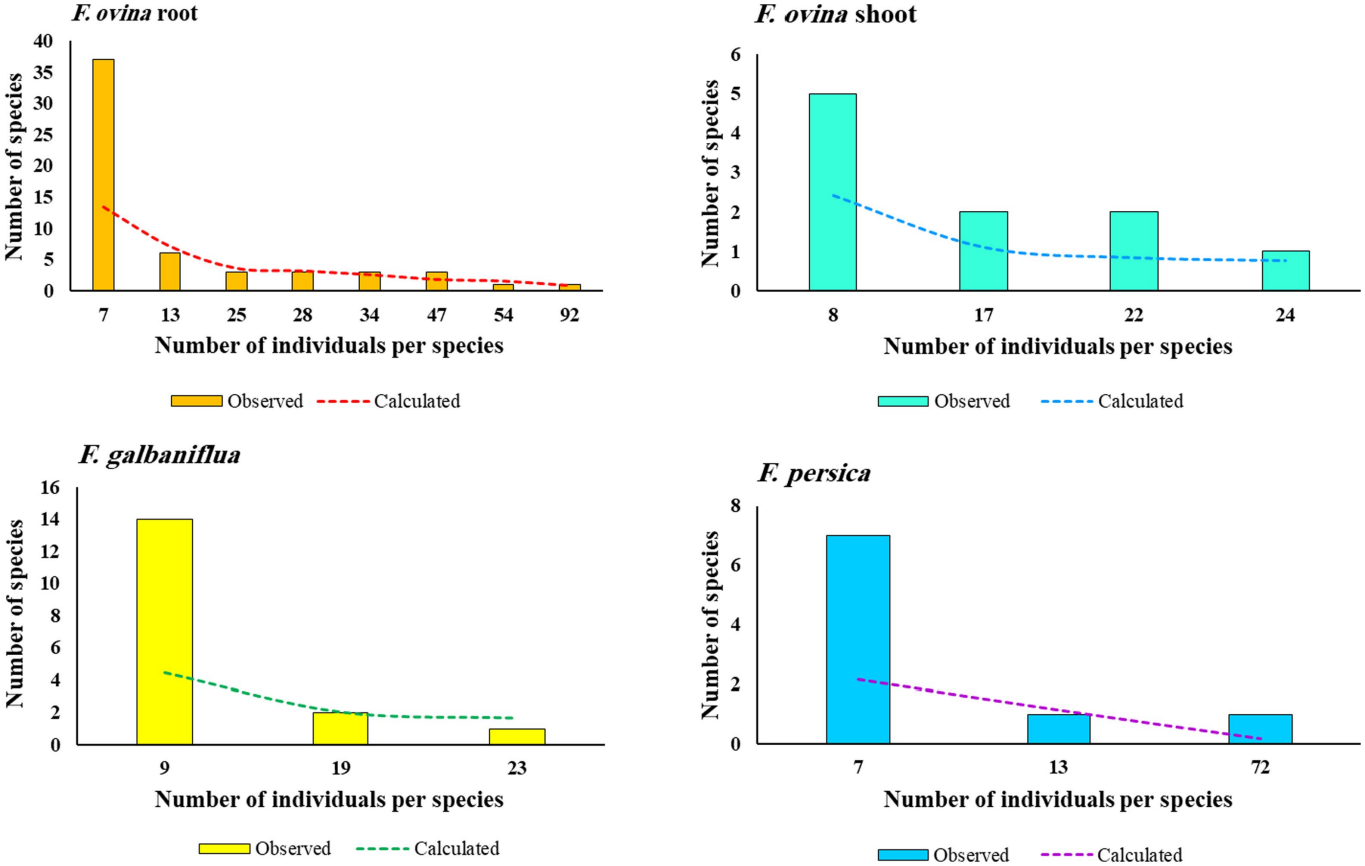
Species frequency-abundance distribution for fungal endophytic strains isolated from *Ferula ovina* roots and shoots, as well as *F. galbaniflua* and *F. persica* roots.

As shown in Figure 5, species frequency/abundance distribution for fungal endophytic strains isolated from *F. ovina*, *F. galbaniflua,* and *F. persica* conformed to the Poisson series, making a curve that resembled a hyperbola. Fisher alpha is a useful parametric index for log-series models of distribution. Fisher alpha for endophytic fungi isolated from *F. ovina* roots, *F. ovina* shoots, *F. galbaniflua* roots, and *F. persica* roots was estimated at 13.6, 2.45, 4.54, and 2.17, respectively (Table 1). As shown by Fisher alpha, the diversity of endophytic fungi isolated from *F. ovina* roots was highest, followed by *F. galbaniflua* roots.

**Table 1:** Diversity indices calculated for the endophytic fungal species community isolated from *F. ovina*, *F. galbaniflua*, and *F. persica.*

Diversity indices	*F. ovina* roots	*F. ovina* shoots	*F. ovina* roots and shoots	*F. galbaniflua* roots	*F. persica* roots
Chao richness estimator	152.14	13.33	130	47.33	19.50
Margalef diversity index	8.25	1.82	8.51	3.06	1.63
Shannon–Wiener diversity index	3.67	2.20	3.71	2.77	1.64
Simpson dominance index (*D*)	0.036	0.121	0.034	0.068	0.317
Simpson diversity index (1-*D*)	0.963	0.879	0.966	0.932	0.683
Reciprocal of Simpson index (1/*D*)	27.45	8.26	29.48	14.66	3.15
Berger–Parker dominance index	0.104	0.169	0.090	0.123	0.537
Reciprocal of Berger–Parker index	9.62	5.92	11.16	8.13	1.86
Pielou evenness index	0.907	0.954	0.907	0.976	0.746
Menhinick index	1.92	0.84	1.87	1.24	0.78
Camargo index	0.522	1.192	0.534	0.804	1.286
Evenness_(e^*H*′)/*S*	0.687	0.900	0.683	0.936	0.572
Fisher_alpha	13.60	2.45	13.90	4.54	2.17

### 3.3 Species richness

Supplementary Table S3 presents the total number of species, *S*, a direct measurement of species diversity. The maximum species number was isolated from *F. ovina*, followed by *F. galbaniflua* roots and the lowest species number was recorded for *F. persica* roots. Regarding the total number of individuals, the same trend was observed, so the highest number of endophytic fungi was isolated from *F. ovina* (Supplementary Table S3). *F. ovina* roots had the lowest Berger–Parker dominance index, followed by *F. galbaniflua* roots. Berger–Parker index and its reciprocal showed that the highest dominance and lowest diversity were observed in endophytic fungi isolated from *F. persica*, followed by *F. ovina* shoots (Table 1). Chao1 index showed that the endophytic fungal community isolated from *F. ovina* roots had the highest species abundance (152.14) and *F. ovina* shoots (13.33) the lowest, followed by *F. persica* roots (19.50). The total number of species, S, depends on the sample size; therefore, it has limitations as a comparative index. Margalef and Menhinick indices, popular sample size-independent indices, were calculated to measure species richness. The values of these indices were higher for endophytic fungi isolated from *F. ovina* roots compared with those from *F. ovina* shoots, *F. galbaniflua,* and *F. persica* roots (Table 1).

### 3.4 Species evenness

Species equitability or evenness—that is, the individual distribution pattern among the species—must be considered when studying species diversity. In other words, the endophytic community composition can be characterized by the dominance and evenness indices. The term “evenness” describes the distribution of species abundance in a community. The evenness index would peak at one when all species in the sample are equally plentiful and would progressively decline toward 0 as the RAs of the species deviate from evenness. The calculated evenness (*E*) values were 0.69, 0.90, 0.94, and 0.57 for endophytic fungi isolated from *F. ovina* roots, *F. ovina* shoots, *F. galbaniflua* roots, and *F. persica* roots, respectively, indicating the species distribution was tilted toward evenness. Pielou evenness index (J) values displayed resemble trend, 0.91, 0.95, 0.98, and 0.75 for endophytic fungi isolated from *F. ovina* roots, *F. ovina* shoots, *F. galbaniflua* roots, and *F. persica* roots, respectively. Pielou index suggested that *F. galbaniflua* roots have the highest value of fungal community evenness (0.98) and *F. persica* the lowest (0.75). The complementarity of Simpson diversity index (1-*D*) values was estimated at 0.96, 0.88, 0.93, and 0.68 for fungi isolated from *F. ovina* roots, *F. ovina* shoots, *F. galbaniflua* roots, and *F. persica* roots, respectively. Host-specific fungal species dominance calculated using the Camargo index was the highest in *F. persica* roots (1.29), followed by *F. ovina* shoots (1.19). The Camargo index for endophytic fungal species isolated from *F. ovina* roots, *F. ovina* (roots and shoots), and *F. galbaniflua* roots was estimated at 0.52, 0.53, and 0.80, respectively (Table 1). As the relative frequency of all endophytic fungal species isolated from *F. ovina*, *F. galbaniflua*, and *F. persica* was less compared with their estimated Camargo index (Table 1; Supplementary Figures S9–S11), no dominant fungal species were observed in these *Ferula* species. The distribution of species within the fungal community isolated from *F. ovina* roots was extremely even, as indicated by the low ecological dominance. The value of the Simpson dominance index (*D*) ranges from 0 to 1. If the index value is close to zero, it means that there is no dominant species, and it shows community stability. Meanwhile, if the value of the index is close to 1, it means the presence of dominant species and indicates that the ecosystem is in unstable conditions and there is ecological pressure (Oktavianti and Purwanti, 2014). The Simpson dominance index (*D*) for the endophytic fungal community of three *Ferula* species was close to zero, so it can be concluded that the fungal community structure for three *Ferula* species is stable and there is no ecological pressure.

**Figure 6:**
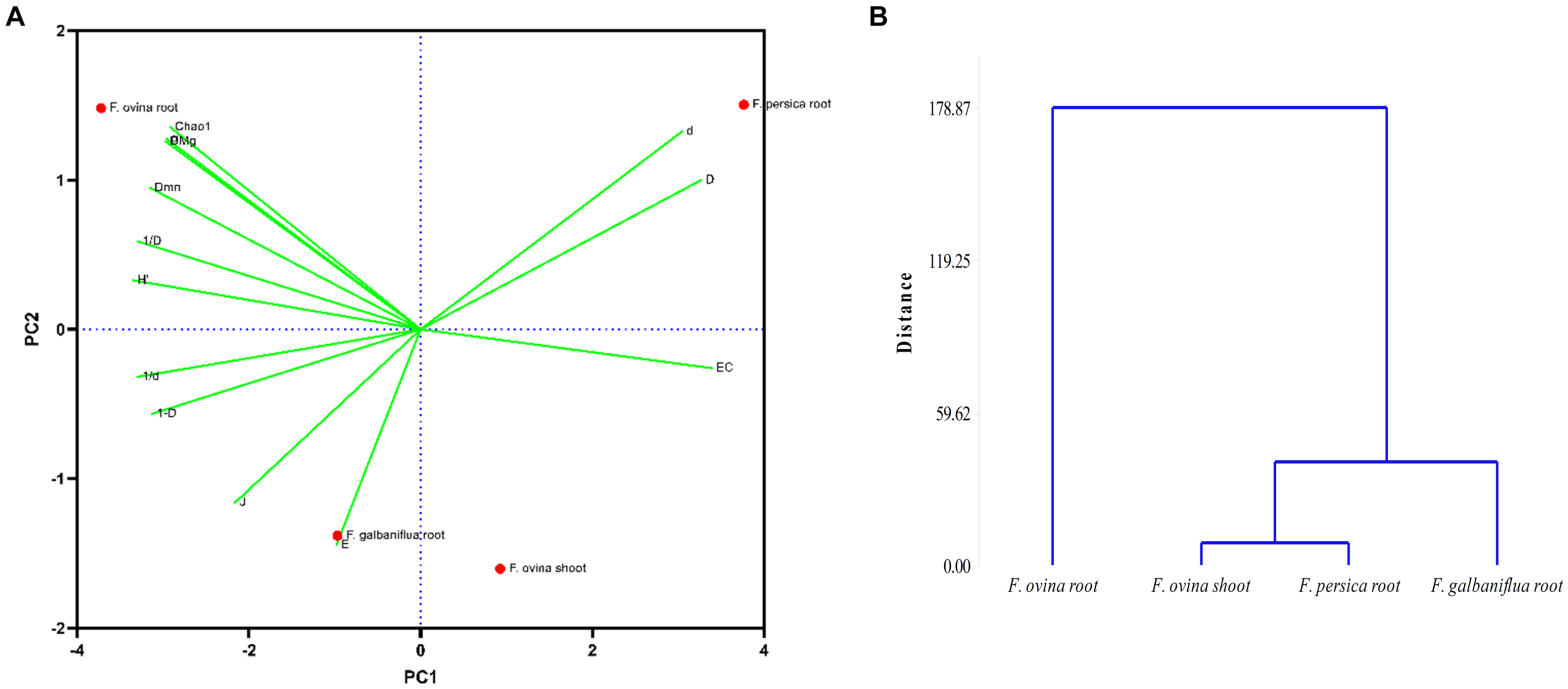
Principal component analysis; the position of *Ferula ovina*, *F. galbaniflua*, and *F. persica* based on diversity indices calculated for the endophytic fungal species community isolated from them **(A)** and the dendrogram of classifying mentioned *Ferula* species based on the diversity indices of their endophytic fungal species community **(B)**. Chao1, Chao richness estimator; *D*_Mg_, Margalef diversity index; *H*′, Shannon–Wiener diversity index; *D*, Simpson dominance index; 1-*D*, Simpson diversity index; 1/*D*, Reciprocal of Simpson index; *d*, Berger–Parker dominance index; 1/*d*, Reciprocal of Berger–Parker index; *J*, Pielou evenness index; *D_mn_*, Menhinick index; *EC*, Camargo index; *E*, Evenness_(e^*H*′)/*S*; *α*, Fisher alpha.

Principal components analysis was carried out on *F. ovina* shoots and roots. *F. galbaniflua* and *F. persica* roots showed that the two first principal components explained 99.37% of the total variation, indicating an excellent fit of the data to the model (Figure 6). The first principal component, which explained 76.46% of the variation, has a high positive coefficient for Camargo, Simpson dominance indices, and Berger–Parker dominance, respectively (Figure 6). The second principal component explained 22.91% of the total variation. The coefficients of the eigenvector of the second principal component showed that the weight of the Chao richness estimator had a high positive coefficient (Figure 6). According to the results, the first and second principal components can be named as effective factors in dominance and richness, respectively. As shown in Figure 6, the endophytic fungal community isolated from *F. persica* roots had the highest species dominance, and endophytic fungi of *F. ovina* roots displayed the highest species richness and subsequently the highest species diversity (Figure 6).

### 3.5 Extrapolation of fungal endophytic species diversity of *F. ovina*, *F. galbaniflua*, and *F. persica*

A small number of fungal species are typified by many colony-forming units (CFUs), whereas many species are typified by few CFUs. Species frequency/abundance distribution presents the fundamental pattern of niche usage in the community. Plotting the different abundance of fungal endophytic species in the community according to species frequency, from most abundant to least abundant, revealed the distinctive distribution of the fungal species that made up the endophytic fungal community of *F. ovina*, *F. galbaniflua*, and *F. persica*. This blatantly displays a log-normal distribution (Figure 5), suggesting that the interaction of numerous independent environmental factors controls multiplicatively the abundances of the fungal species (Heip et al., 1998).

The frequency of species with different abundance was calculated by the Fisher series for endophytic fungi isolated from three *Ferula* species (dotted line in Figure 5). The affinity in the observed and calculated series for fungal endophytes isolated from *F. ovina* roots, except number of species with the least abundance, was very striking. However, the fit for the fungal endophytes isolated from *F. galbaniflua* roots, *F. persica* roots, and *F. ovina* shoots was not good. There were comparatively more rarer species and fewer commoner ones, compared with the calculated series, for fungal endophytes isolated from *F. galbaniflua* roots. Such results were likewise reported by Fisher et al. (1943).

### 3.6 Beta diversity analysis of endophytic fungi from *F. ovina*, *F. galbaniflua,* and *F. persica*

Different ecosystems are generally home to various biological communities. Beta diversity, introduced by Whittaker (1960), is defined as differences in species composition among ecosystems within an area of interest. Sorensen (β_Sor_; Sorensen, 1948) and Jaccard (β_Jac_; Jaccard, 1912) indices are the two most widely used non-parametric beta diversity ones. Values of the Jaccard and Sorensen classic indices showed that *F. ovina* roots had the most similarity in endophytic fungal species with *F. galbaniflua* roots and then *F. ovina* shoots (Table 2). The fungal endophytes isolated from *F. ovina* roots belonged to 57 species (Supplementary Figure S8), 49 species were root-specific (not isolated from *F. galbaniflua* roots), and 8 species were common with 17 fungal species isolated from *F. galbaniflua* roots (Supplementary Figure S8). Ten endophytic fungal species were isolated from *F. ovina* shoots; three fungal species were shoot-specific, but seven of them were also isolated from its roots. As presented in Table 2, *F. ovina* shoots displayed the highest similarity with its roots in terms of endophytic fungal species. No similarity was observed between endophytic fungal species isolated from *F. persica* roots than those isolated from *F. galbaniflua* roots, and *F. ovina* shoots (Table 2).

**Table 2:** Jaccard (above the diameter) and Sorensen (bottom diameter) similarity indices between the fungal species community isolated from *Ferula ovina*, *F. galbaniflua*, and *F. persica.*

Tissue	*F. ovina* roots	*F. ovina* shoots	*F. ovina* roots and shoots	*F. galbaniflua* roots	*F. persica* roots
*F. ovina* roots	–	0.1167	–	0.1212	0.0154
*F. ovina* shoots	0.2090	–	–	0.0800	0.0000
*F. ovina* roots and shoots	–	–	–	0.1159	0.0147
*F. galbaniflua* roots	0.2162	0.1481	0.2078	–	0.0000
*F. persica* roots	0.0303	0.0000	0.0290	0.0000	–

### 3.7 Community ordination and classification

**Figure 7:**
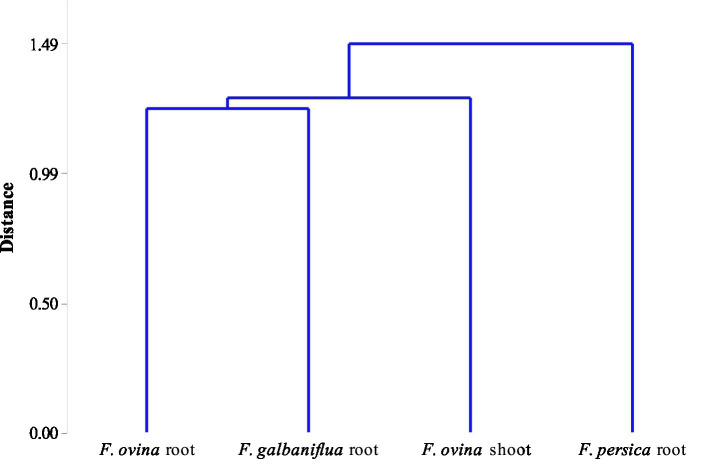
Dendrogram of classifying *Ferula ovina*, *F. galbaniflua*, and *F. persica* based on the Jaccard similarity index of their endophytic fungal species community.

Realizing the natural system that has resulted in the observed grouping of species depends critically on community classification. There are several characteristics shared by all the fungal species in the group that help to distinguish them from other group(s). Cluster analysis was used to classify *Ferula* spp. based on the Jaccard similarity index of their endophytic fungal species community. *F. ovina* roots and *F. galbaniflua* roots clustered together, and they formed a group with *F. ovina* shoots that was distinct from *F. persica* roots (Figure 7).

### 3.8 Specificity of community composition

There is one common genus of endophytic fungus in *F. ovina*, *F. galbaniflua*, and *F. persica*, indicating that *Acremonium* is the main genus that puts together the culturable fungal community structure of the three mentioned *Ferula* species (Figure 4). Concurrently, the endemic genera isolated from different *Ferula* species showed that host genetics might display a remarkable effect on the fungal community structure inhabiting *Ferula* spp.

**Figure 8:**
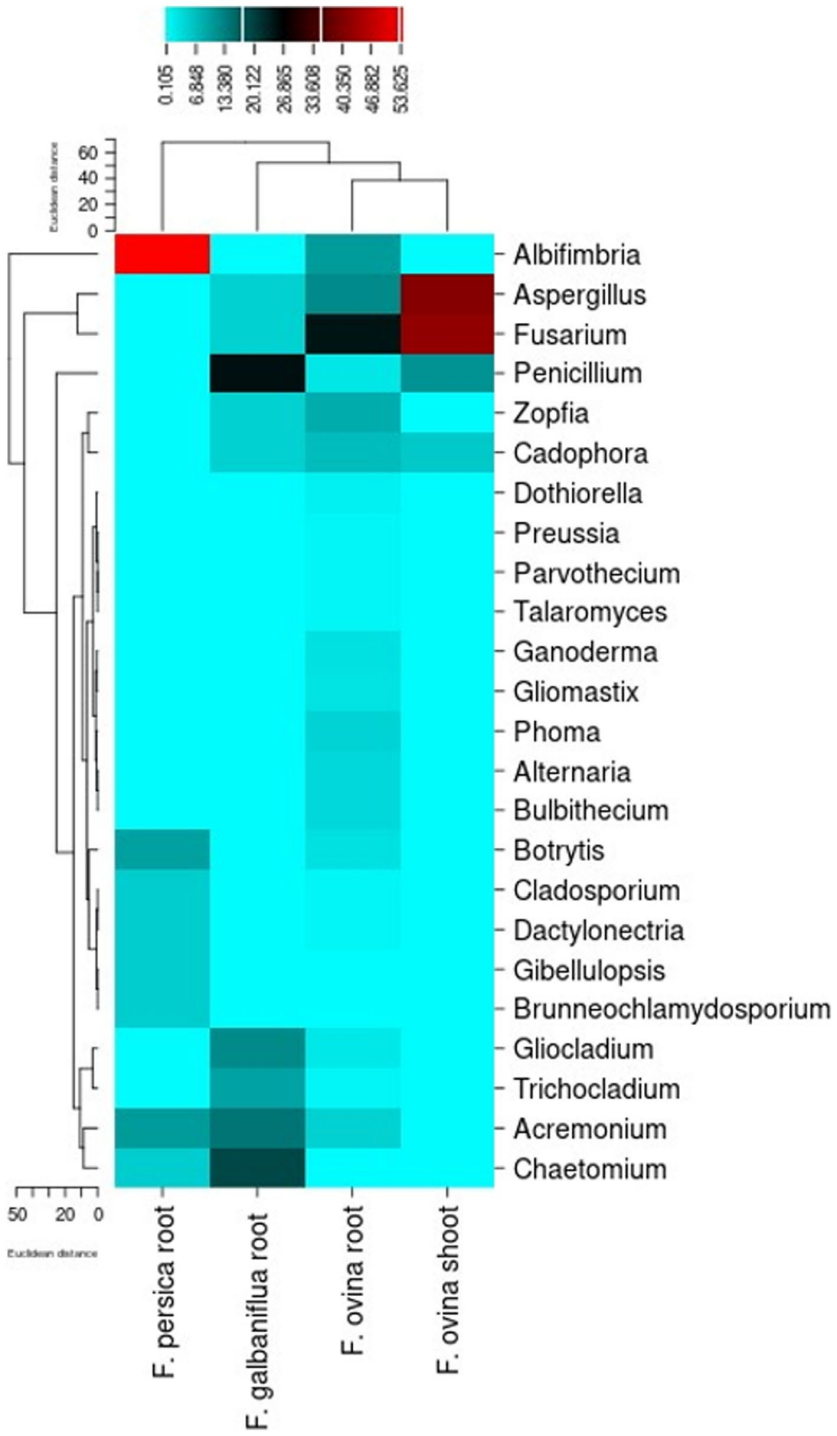
Heat map indicating the relative frequency of the endophytic fungal genera isolated from *Ferula ovina*, *F. galbaniflua*, and *F. persica.*

The different endophytic fungal genera isolated from *F. ovina*, *F. galbaniflua,* and *F. persica* were classified based on their relative frequency (Figure 8). A genus-level heatmap was made to evaluate possible trends of the relative frequency of endophytic fungal genera by the host (Figure 8); no unidentified fungi were presented in this graph. *Fusarium* displayed the most abundant genus in *F. ovina* roots and shoots. *Penicillium* and *Albifimbria* were the most abundant genera in *F. galbaniflua* roots and *F. persica* roots, respectively (Figure 8).

The various *Ferula* species were arranged regarding the axes so that relative location to the axes and one another maximized the information on the diversity of endophytic fungal genera isolated from *Ferula* spp. (Supplementary Figure S12). Principal component analysis performed on 24 endophytic fungal species indicated that the first two principal components explained 80.98% of dataset variation (Supplementary Figure S12). The first and second principal components accounted for 48.42 and 32.56%, respectively, of the total variation and distinguished fungal genus assemblages among *F. ovina* roots and shoots, *F. galbaniflua* roots, and *F. persica* roots. The highest RA of *Albifimbria*, *Botrytis*, *Cladosporium*, *Gibellulopsis*, *Brunneochlamydosporium*, and *Dactylonectria* belonged to *F. persica* roots (Supplementary Figure S12). As shown in Supplementary Figure S12, *F. ovina* shoots and *F. galbaniflua* roots displayed the most similarity in terms of relative endophytic fungal genera. The relative frequency of *Zopfia* and *Bulbithecium* in *F. ovina* roots was the highest compared with *F. ovina* shoots, *F. galbaniflua,* and *F. persica* roots. *Alternaria*, *Bulbithecium*, *Phoma*, *Dothiorella*, *Parvothecium*, *Talaromyces*, *Ganoderma*, *Gliomastix*, and *Preussia* only colonized the roots of *F. ovina* roots (Supplementary Figure S12).

### 3.9 Occurrence percentage of endophytic fungal species in *F. ovina*, *F. galbaniflua*, and *F. persica*

Based on the percentage of occurrence of endophytic fungi—the proportion of *Ferula* species from which each endophytic fungal species was isolated to the number of studied *Ferula* spp.—they were grouped somewhat arbitrarily in this study. There were three categories; fungal species with 1–33%, 34–67%, and 68–100% were considered sporadic, occasional, and common, respectively. Although this classification is not perfect, it was believed to shed light on the fungal species occurrence in *Ferula* spp. and simplify the description. Although some fungal species were common members of the mycoflora of two *Ferula* species studied, each *Ferula* species was found to have a unique mycoflora. Overall, many endophytic fungal species (88.31%) were sporadically (33%; one of three species) in *Ferula* spp. Only a smaller number of fungal species (11.69) were isolated occasionally (67%; two of three species) from *Ferula* species.

## 4 Discussion

The present study is unique because no particular research has been conducted on the fungal species diversity inhabiting the tissues of *Ferula* spp. Furthermore, this is the first report on fungal communities isolated from *Ferula* spp. growing naturally in Iran. Fungal communities exhibit geographical heterogeneity, which has been attributed to factors including host domain range, dispersion limitations, environmental selection, and regional adaptation development. The endophytic community is mostly affected by rainfall. The dispersion of fungal spores and the colonization of endophytes may be aided by rainfall, and high humidity plays an important role in spore germination. Furthermore, a moderate annual temperature may prolong the fungal lifespan and boost the colonization rate of endophytic fungi (Peay et al., 2010).

The results indicated that a range of fungal species are symbiotic with *Ferula* spp.; all 1,348 isolates were morphologically and molecularly classified into 77 species, and the majority of isolates fell into the phylum of Ascomycetes (Figures 1–3). *F. ovina*, *F. galbaniflua,* and *F. persica* exhibited different patterns in terms of the presence of endophytic fungal species, suggesting a diversity of host-specific endophytic fungi. The diversity of host-specific endophytic microbiomes has been previously reported (Singha et al., 2021). Such exclusive distribution of endophytic fungal species in different plant hosts may indicate the special niche requirements of the endophytic species as well as their specific functional role in their host, including promoting the growth and development, tolerating the environmental stress, and protecting against the herbivores and phytopathogens (Nourian et al., 2024; Ranjbar et al., 2024; Salehi and Safaie, 2024).

The fungal frequency and diversity indices in the shoots and roots of *F. ovina*, *F. galbaniflua,* and *F. persica* showed that the endophytic fungal frequency in the roots was remarkably higher compared to that in the shoots. The roots are in direct contact with the soil as a microbial reservoir. Additionally, the soil medium plays a buffering role against temperature and humidity fluctuations, allowing for the greatest richness of endophytic fungi in the roots. Besides, *Ferula* spp. are herbaceous perennial plants, and their roots have longer durability compared with shoots. It could explain why *F. ovina*, *F. galbaniflua,* and *F. persica* roots had higher fungal diversity and species richness compared with their shoots. Moreover, endophytic fungal frequency and distribution are likely affected by the content of biologically active compounds in tissues. Certain fungal species are able to survive in a specific tissue (Carroll and Petrini, 1983). Endophytic tissue-specific diversity has been previously reported (Fang et al., 2019). The higher CF and fungal species diversity of the roots of these *Ferula* species compared with shoots can also be explained by the transmission route of endophytic fungi in arid and semi-arid areas. Most endophytes reside in roots, belong to class 4 of the non-clavicipitaceous (NC) endophytes, and are horizontally transmitted from one host plant to another (Rodriguez et al., 2009). In arid and semi-arid areas, flora is sporadic and has low density and diversity, but plants have well-developed underground systems that form nutrient-rich resource patches (Zuo et al., 2020). Because there are more hosts and substrates for these fungi to infect, the distribution of expanded root systems and nutritional status in a desert environment may therefore result in higher CF and fungal species diversity in the roots compared with the shoots.

However, there are inevitably inherent biases in both culture-independent and culture-dependent methods of studying fungal diversity (Wijayawardene et al., 2021). In the present study, culture-dependent methods were used to allow these endophytic fungi to be used in co-culture with *Ferula* cell culture for enhancing secondary metabolite production and in bioformulation for coping with biotic and abiotic stress in *Ferula*.

## 5 Conclusion

The internal environments of *Ferula* spp. affected the diversity of endophytic fungi they harbored, indicating host specificity. The investigation of the diversity of endophytic fungi in *Ferula* not only provides fungal endophytic resources for exploiting beneficial relations among endophytic fungi and *Ferula* spp., including the synthesis of bioactive compounds, growth promotion, and plant protection against biotic and abiotic stress but also facilitates the effective artificial cultivation of *Ferula* spp. native to Iran on a large scale and their quality control.

## Data availability statement

The original contributions presented in the study are included in the article/Supplementary material, further inquiries can be directed to the corresponding authors.

## Author contributions

NS: Conceptualization, Methodology, Project administration, Resources, Supervision, Validation, Writing – review & editing. MS: Conceptualization, Data curation, Formal analysis, Investigation, Methodology, Project administration, Software, Validation, Visualization, Writing – original draft, Writing – review & editing. MF: Investigation, Writing – review & editing. SF: Data curation, Formal analysis, Methodology, Software, Validation, Writing – review & editing. SK: Investigation, Writing – review & editing. SA: Investigation, Writing – review & editing. J-LY: Funding acquisition, Writing – review & editing. MN: Funding acquisition, Writing – review & editing.
