# Supplementary material for: Culture-based diversity of endophytic fungi of three species of Ferula grown in Iran
Source: Front Microbiol. 2024 May 23;15:1363158. doi: 10.3389/fmicb.2024.1363158 (PMC11153712; doi:10.3389/fmicb.2024.1363158)
Supplement: Supplementary file 1 [file Table_1.pdf]

Culture-based Diversity of Endophytic Fungi of Three Species of *Ferula* Grown in Iran

Naser Safaie[1*], Mina Salehi[2*], Mona Felegari[1], Siamak Farhadi[3], Samira Karimzadeh[1], Sadegh Asadi[4], Jun-Li Yang[5], Mohammad Reza Naghavi[6*]

[1]Department of Plant Pathology, Faculty of Agriculture, Tarbiat Modares University, Tehran, Iran.

[2]Department of Plant Genetics and Breeding, Faculty of Agriculture, Tarbiat Modares University, Tehran, Iran.

[3]Seed and Plant Improvement Institute, Agricultural Research, Education and Extension Organization (AREEO), Karaj, Iran.

[4]Division of Crop Ecology, Department of Agronomy and Plant Breeding, College of Agriculture and Natural Resources, University of Tehran, P.O. Box: 31587-11167, Karaj, Iran.

[5]CAS Key Laboratory of Chemistry of Northwestern Plant Resources and Key Laboratory for Natural Medicine of Gansu Province, Lanzhou Institute of Chemical Physics, Chinese Academy of Sciences, Lanzhou 730000, China.

[6]Division of Biotechnology, Department of Agronomy and Plant Breeding, College of Agriculture and Natural Resources, University of Tehran, P.O. Box: 31587-11167, Karaj, Iran.

*Corresponding authors:

Naser Safaie (nsafaie@modares.ac.ir)

Mina Salehi (salehi.minasm@gmail.com)

Mohammad Reza Naghavi (mnaghavi@ut.ac.ir)

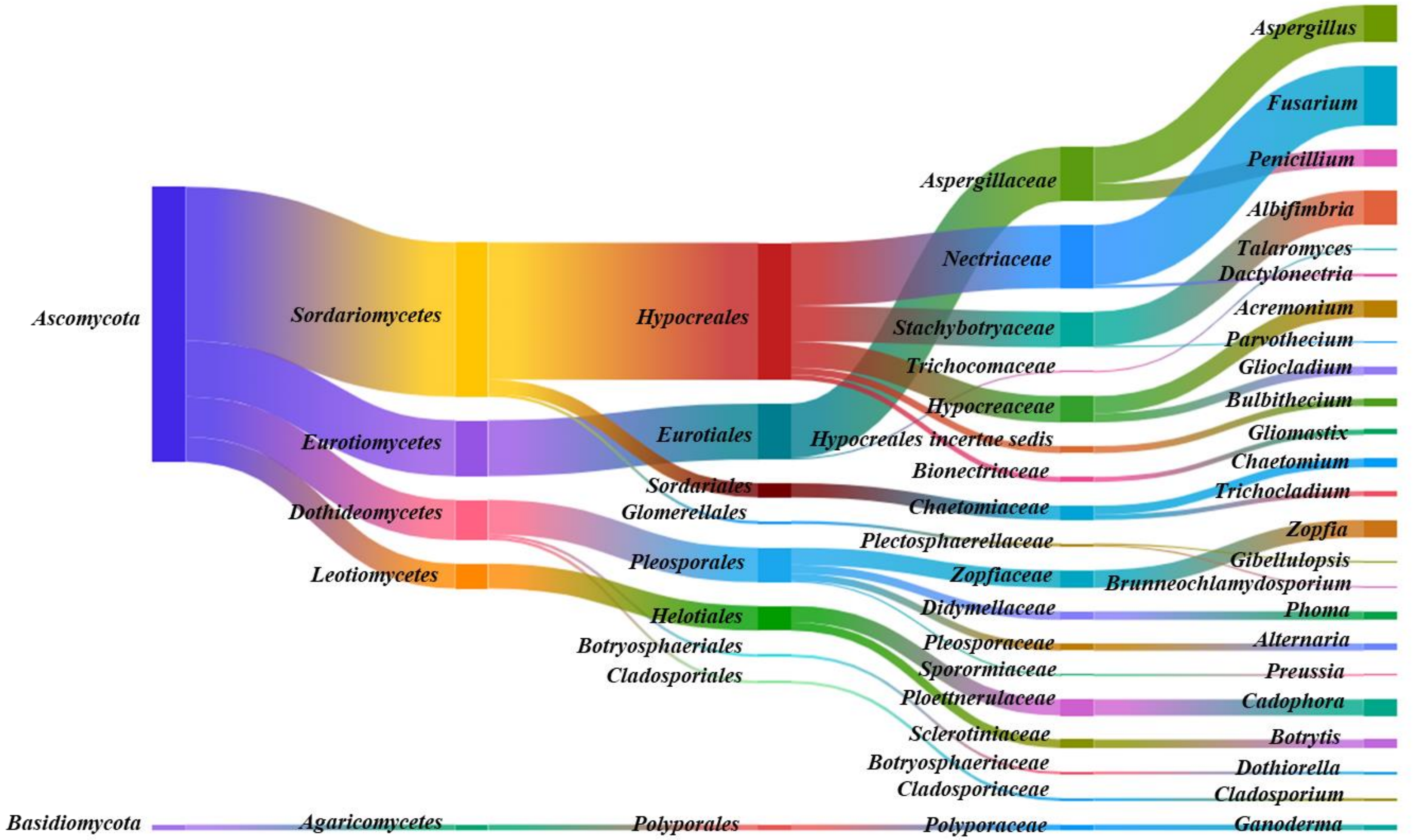

Figure S1. Species taxonomic relationship. The endophytic fungi isolated from *Ferula ovina* shoots and roots, *F. galbaniflua* roots, and *F. persica* roots belonged to two phyla, five classes, nine orders, 18 families, and 24 genera.

Table S1. Number of cultured samples, isolated endophytic fungi and colonized segments, and percentages of colonized segments (colonization frequency) of *Ferula ovina*, *F. galbaniflua*, and *F. persica* tissues

**Tissues**	**No. of samples**	**No. of isolated endophytic fungi**	**No. of colonized segments**	**Colonization frequency**
F. ovina roots	1080	885	360	33.33
F. ovina shoots	1080	142	136	12.59
F. ovina roots and shoots	2160	1027	496	22.96
F. galbaniflua roots	180	187	59	32.78
F. galbaniflua shoots	180	0	0	0
F. galbaniflua roots and shoots	360	187	59	16.39
F. persica roots	360	134	116	32.22
F. persica shoots	360	0	0	0
F. persica roots and shoots	720	134	116	16.11

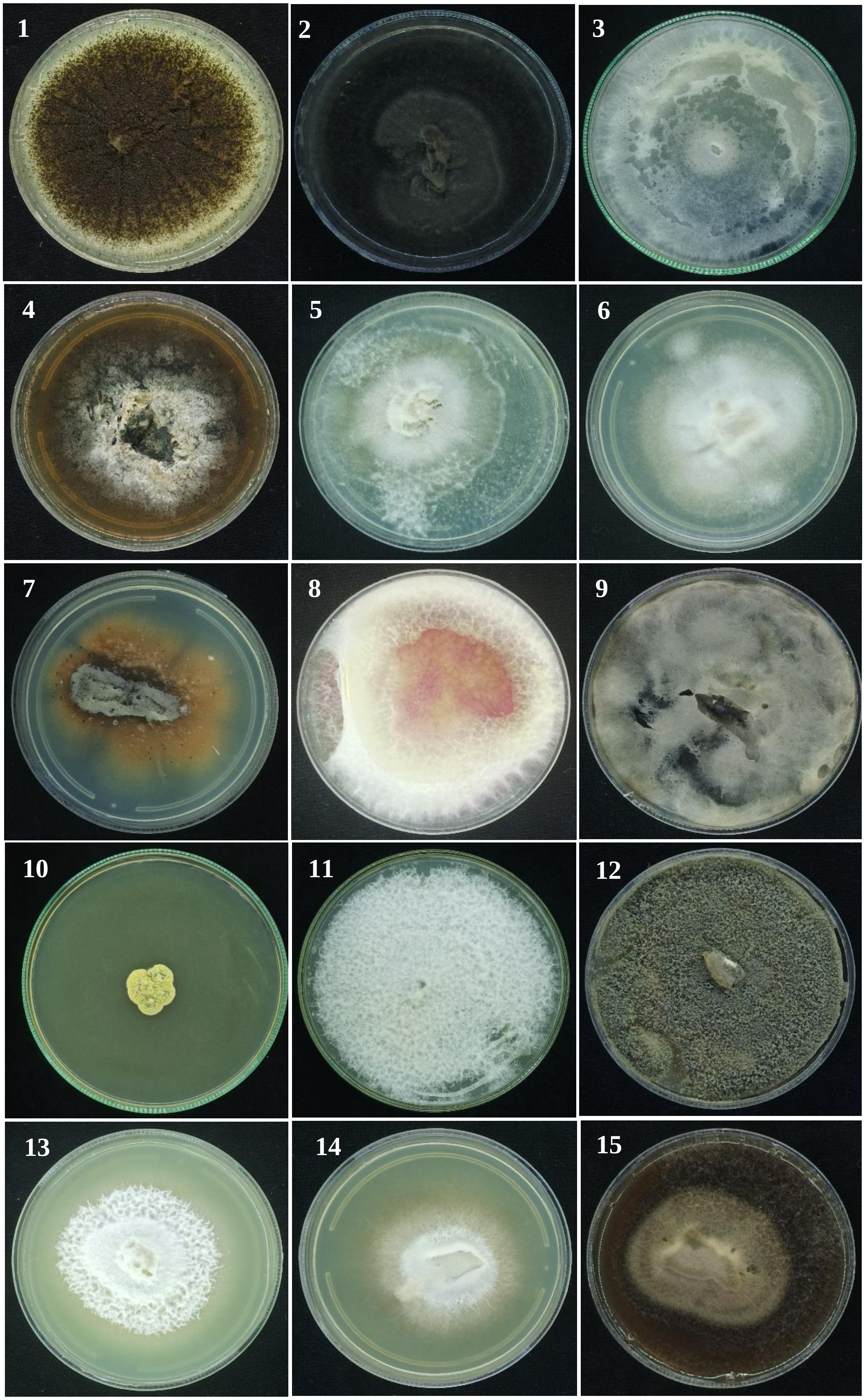
1
2
3
4
5
6
7
8
9
10
11
12
13
14
15

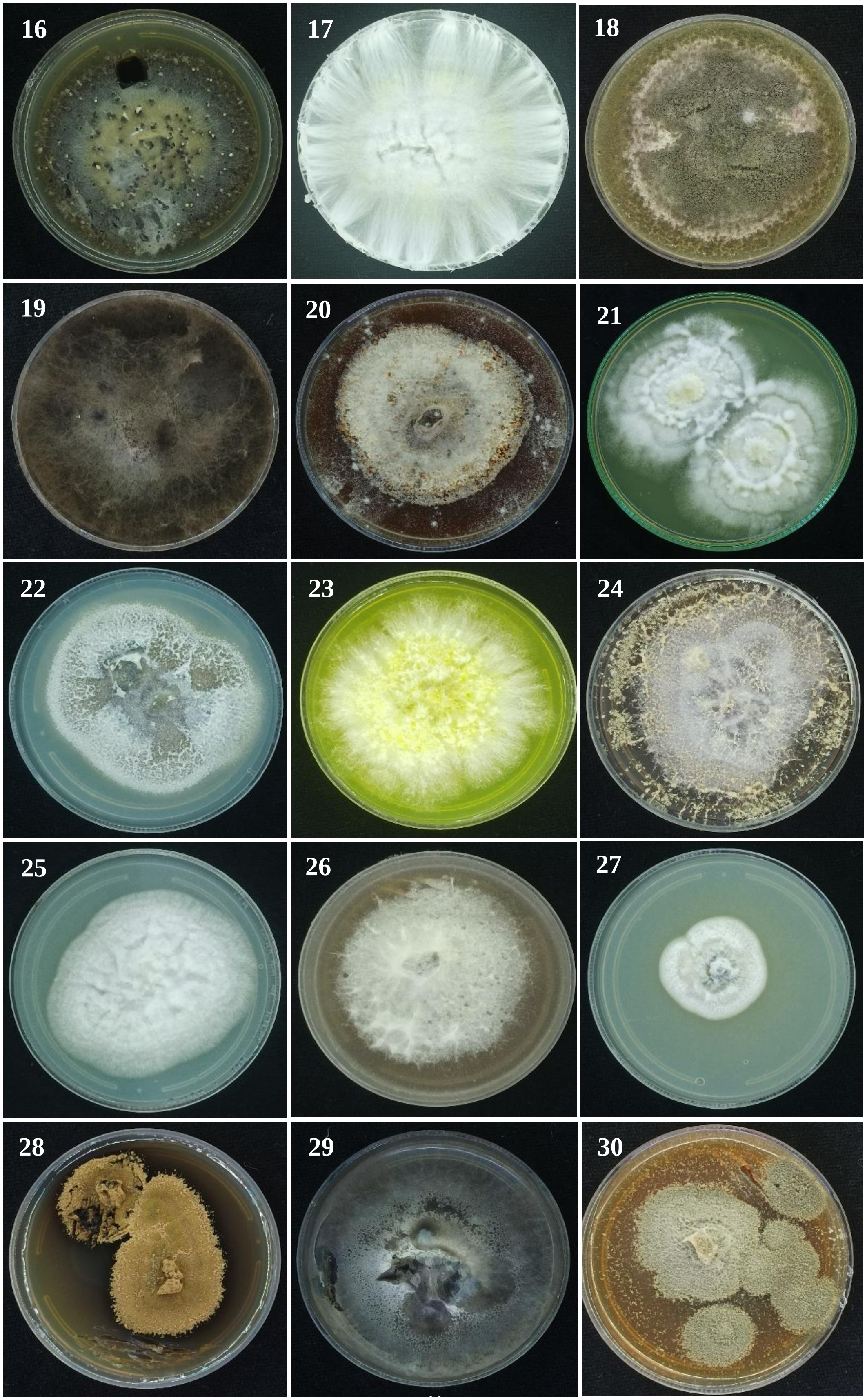
16
17
18
19
20
21
22
23
24
25
26
27
28
29
30

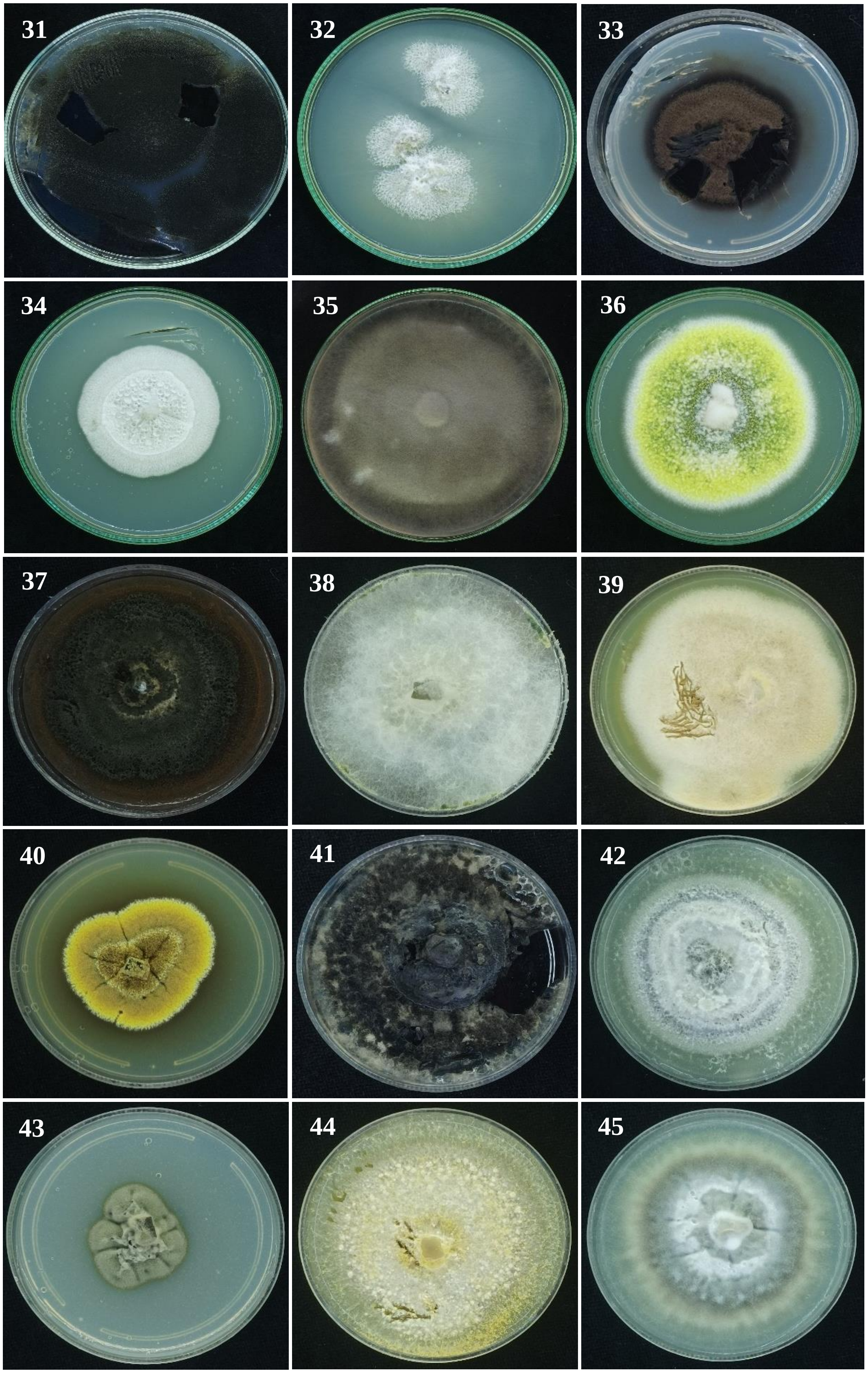
31
32
33
34
35
36
37
38
39
40
41
42
43
44
45

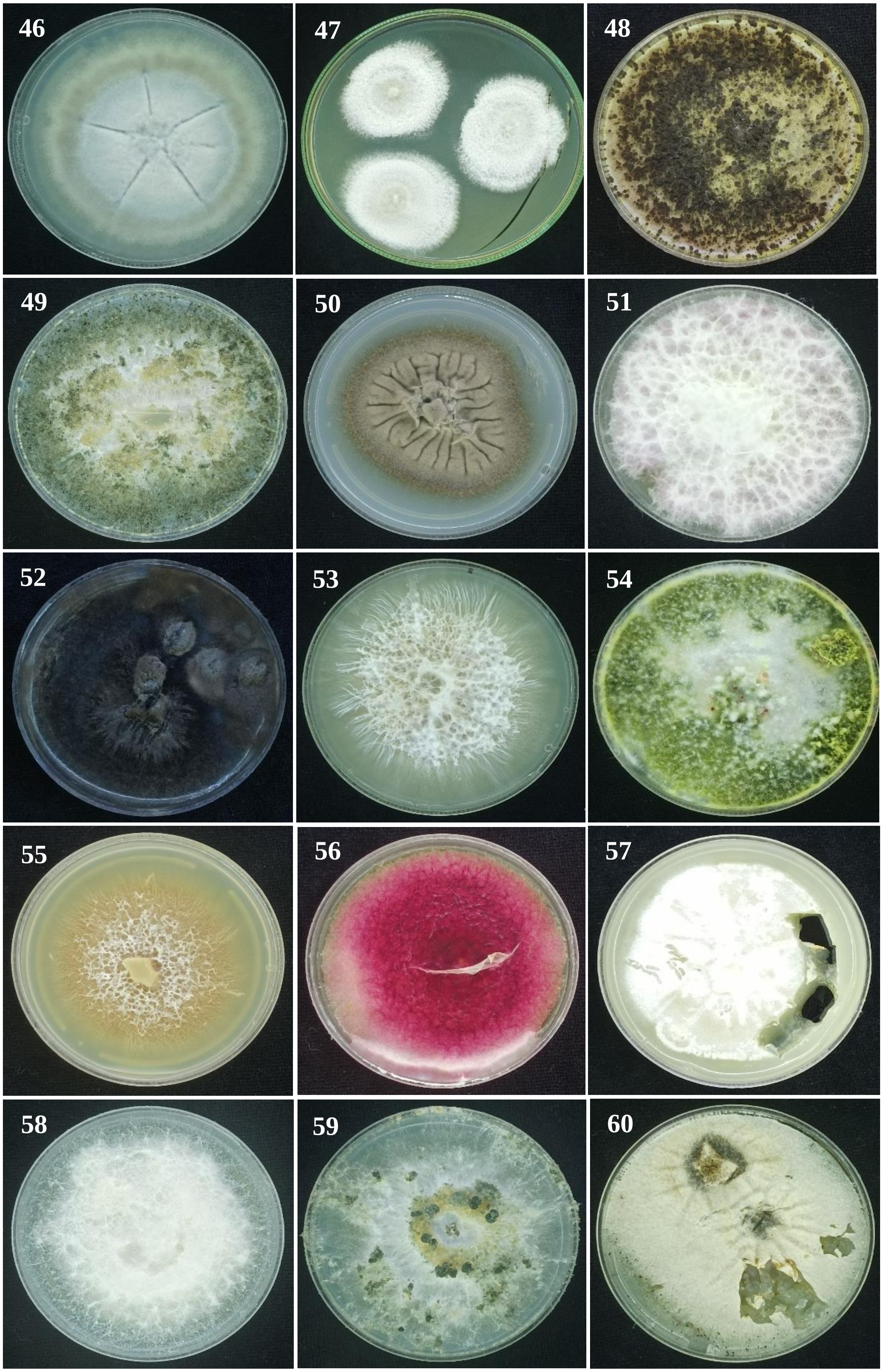
46
47
48
49
50
51
52
53
54
55
56
57
58
59
60

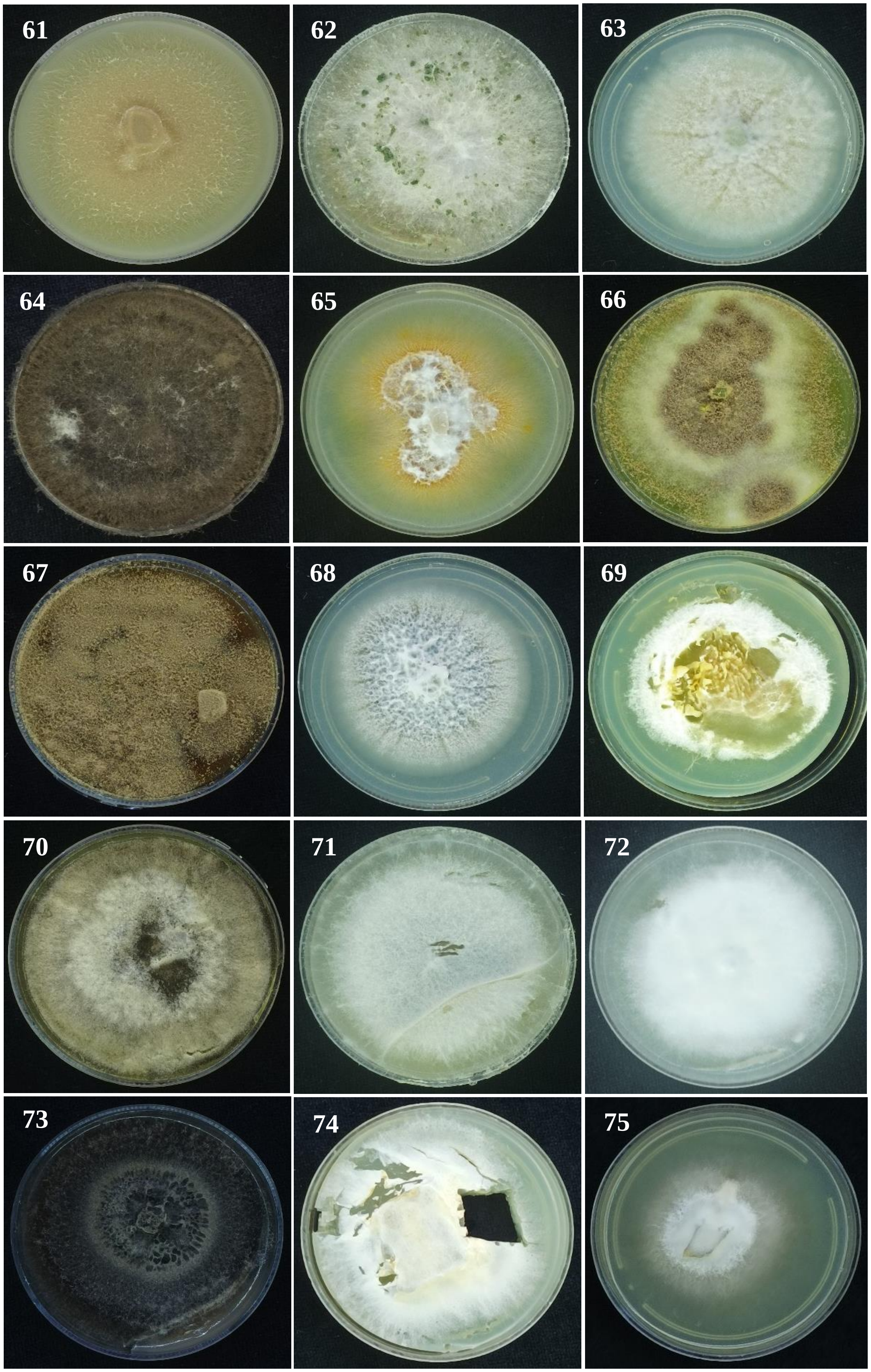
61
62
63
64
65
66
67
68
69
70
71
72
73
74
75

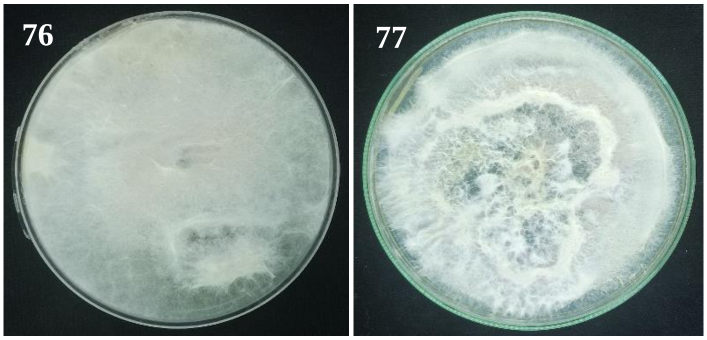

Figure S2. Representative isolates of different morphotypes (77), isolated from *Ferula ovina*, *F. galbaniflua*, and *F. persica* tissues, considered for detailed description and morphological and molecular identification

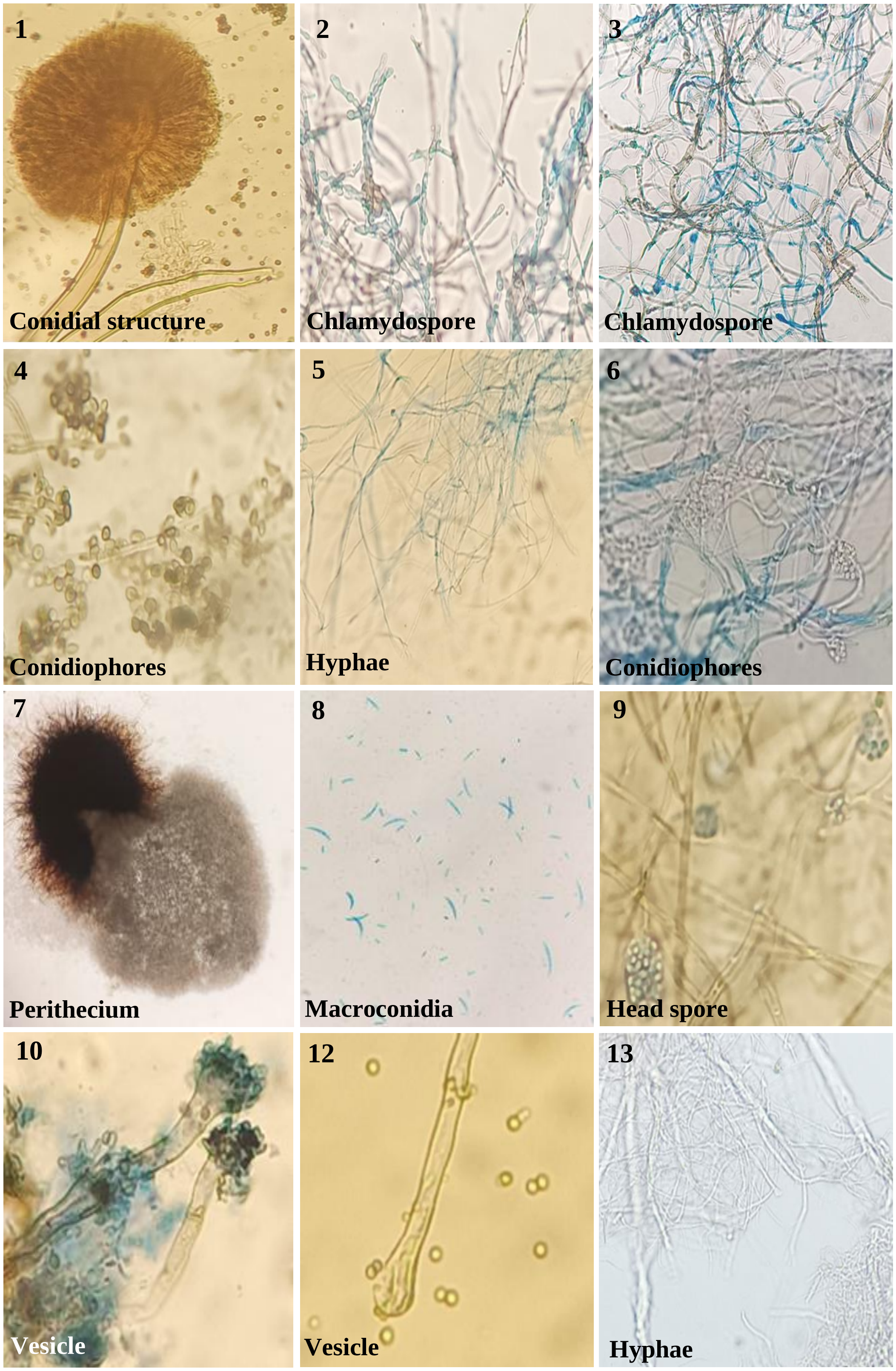
1
Conidial structure
2
Chlamydospore
3
Chlamydospore
4
Conidiophores
5
Hyphae
6
Conidiophores
7
Perithecium
8
Macroconidia
9
Head spore
10
Vesicle
12
Vesicle
13
Hyphae

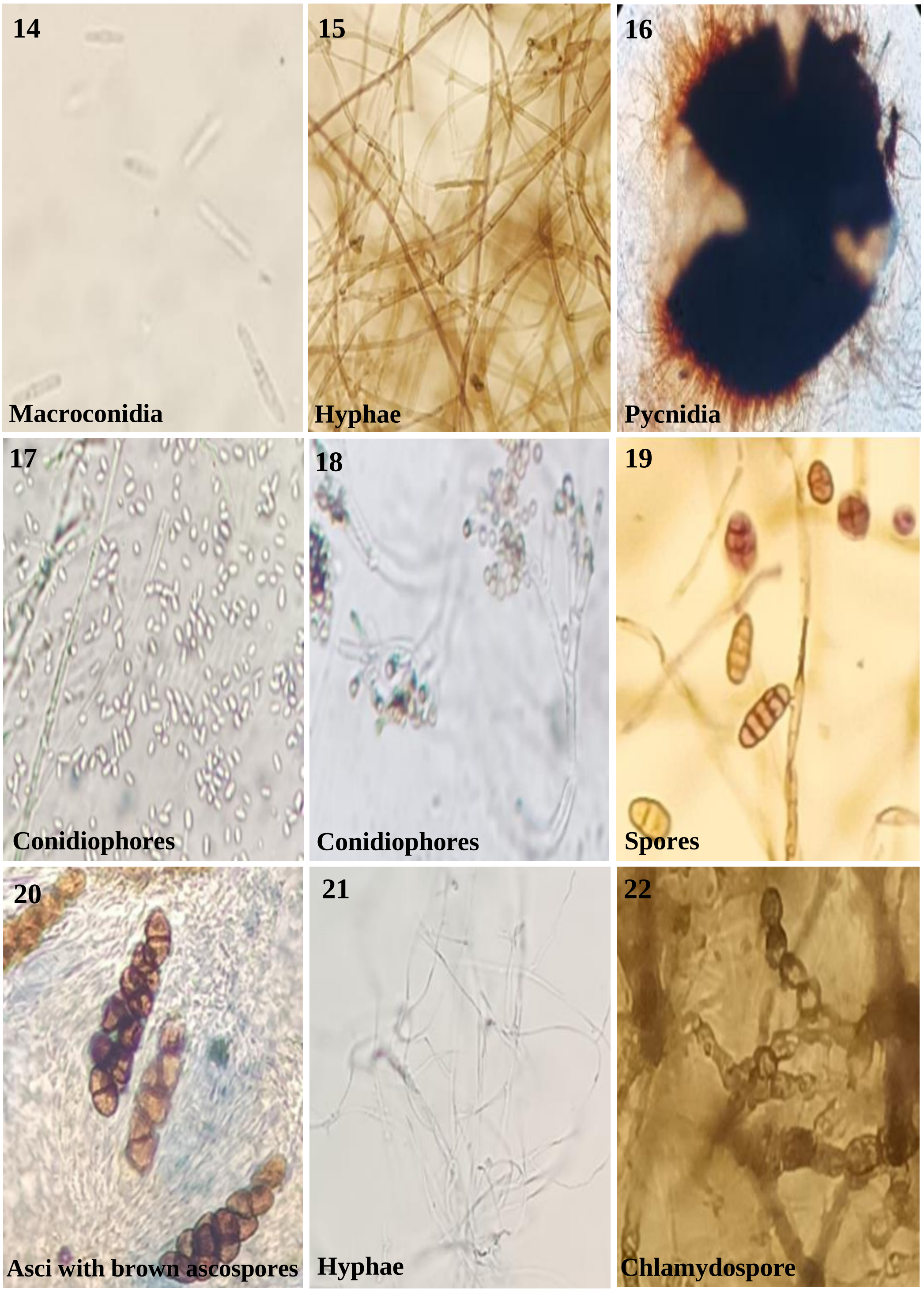
14
Macroconidia
15
Hyphae
16
Pycnidia
17
Conidiophores
18
Conidiophores
19
Spores
20
Asci with brown ascospores
21
Hyphae
22
Chlamydospore

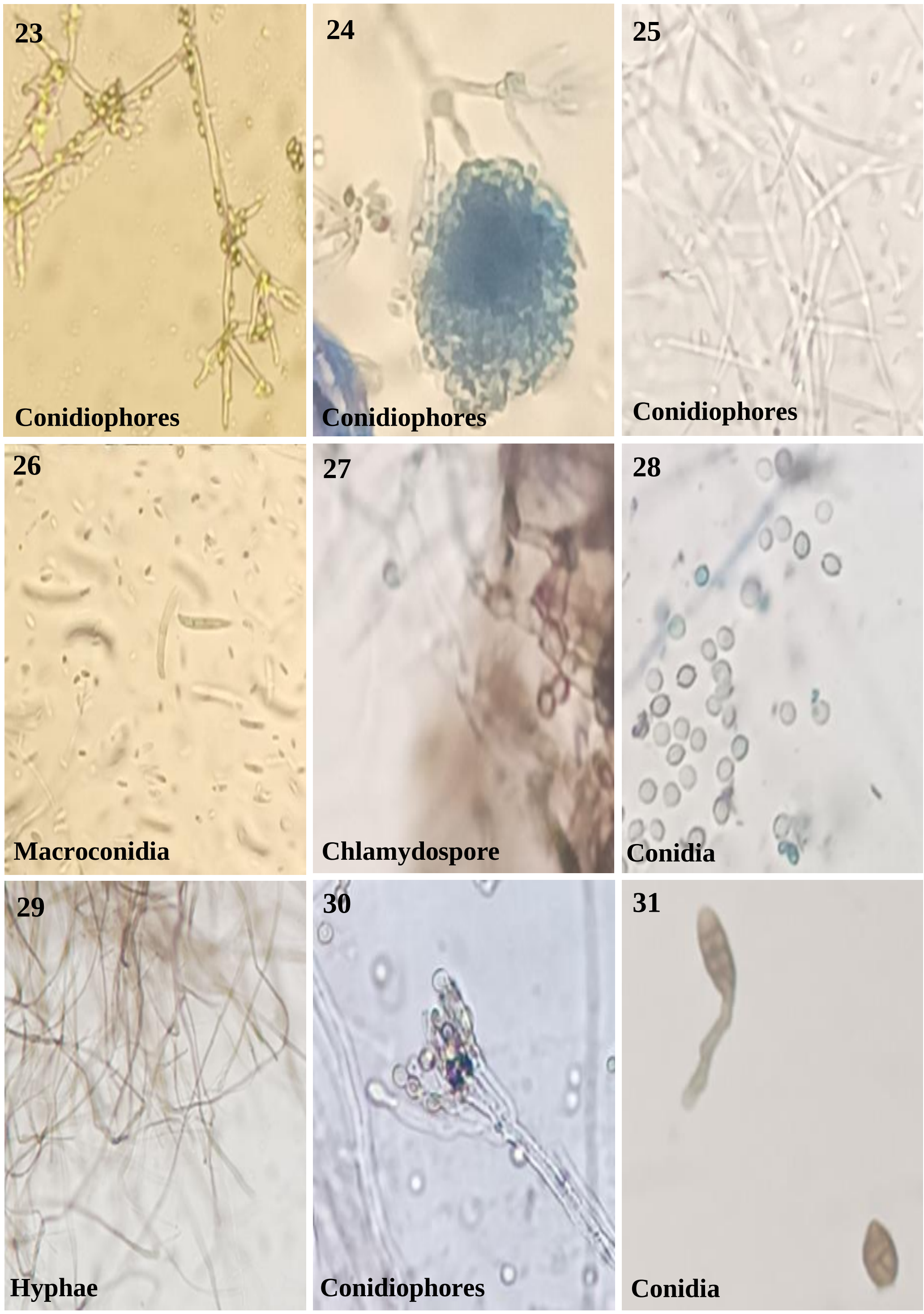
23
Conidiophores
24
Conidiophores
25
Conidiophores
26
Macroconidia
27
Chlamydospore
28
Conidia
29
Hyphae
30
Conidiophores
31
Conidia

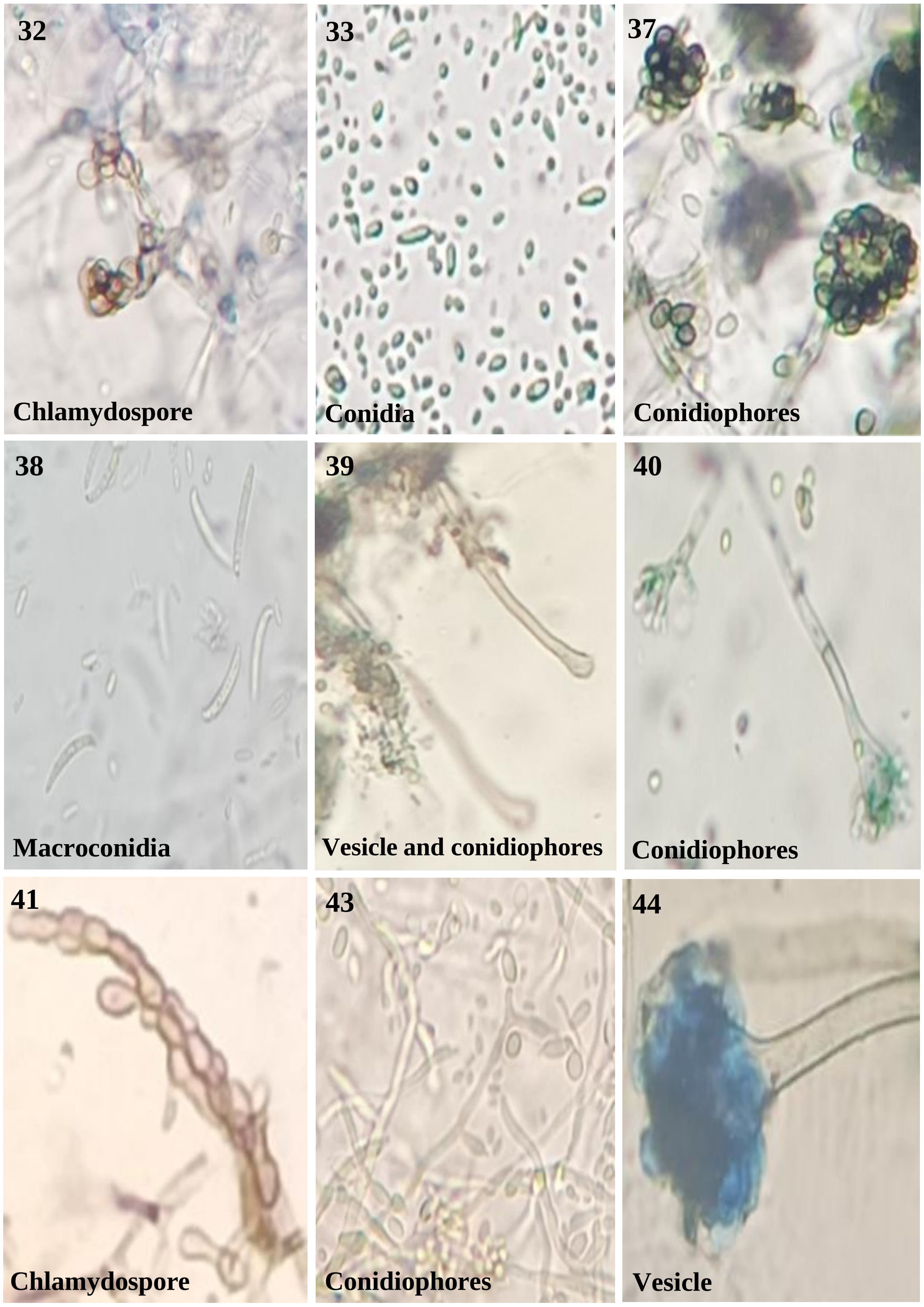
32
Chlamydospore
33
Conidia
37
Conidiophores
38
Macroconidia
39
Vesicle and conidiophores
40
Conidiophores
41
Chlamydospore
43
Conidiophores
44
Vesicle

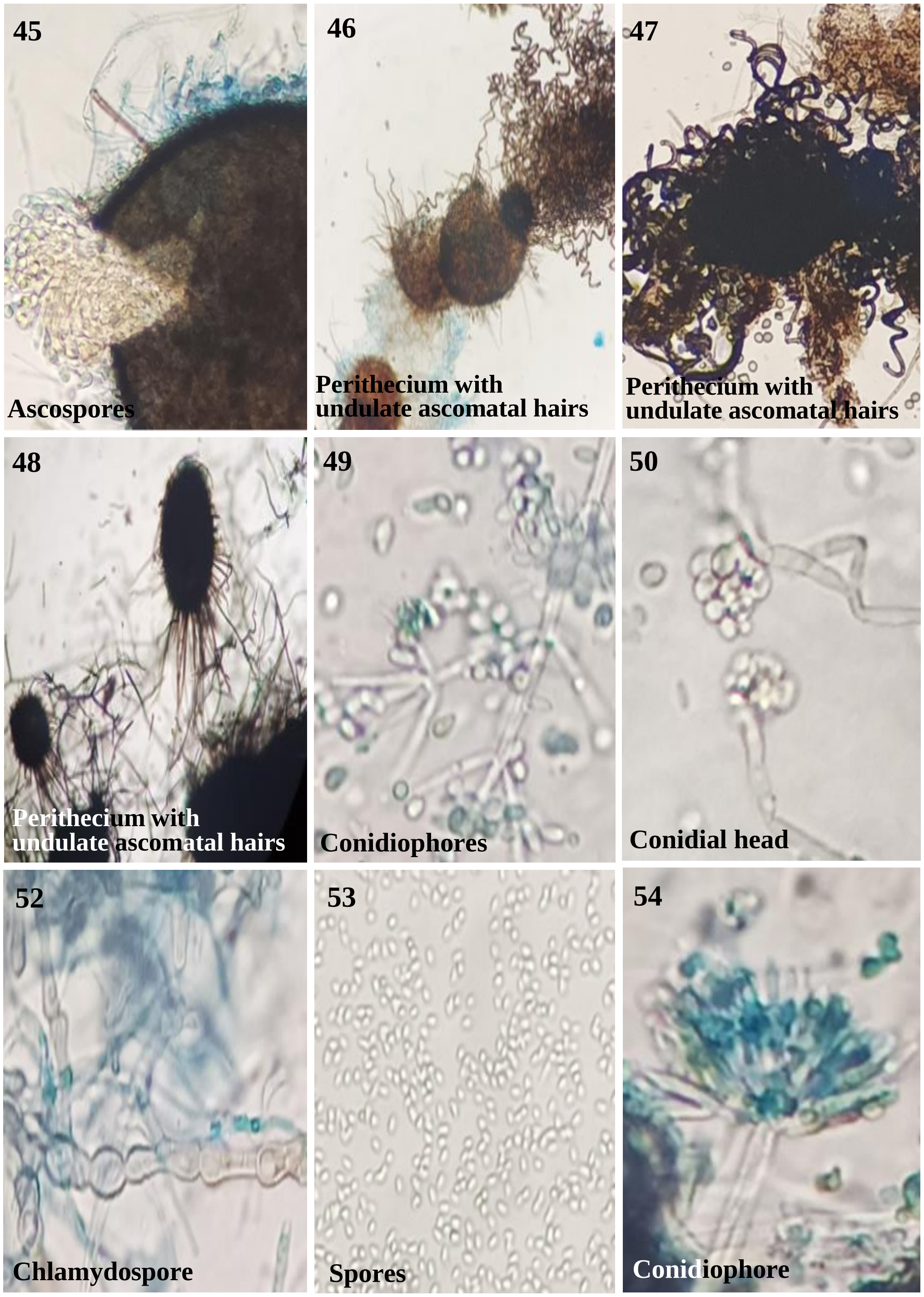
45
Ascospores
46
Perithecium with
undulate ascomatal hairs
47
Perithecium with
undulate ascomatal hairs
48
Perithecium with
undulate ascomatal hairs
49
Conidiophores
50
Conidial head
52
Chlamydospore
53
Spores
54
Conidiophore

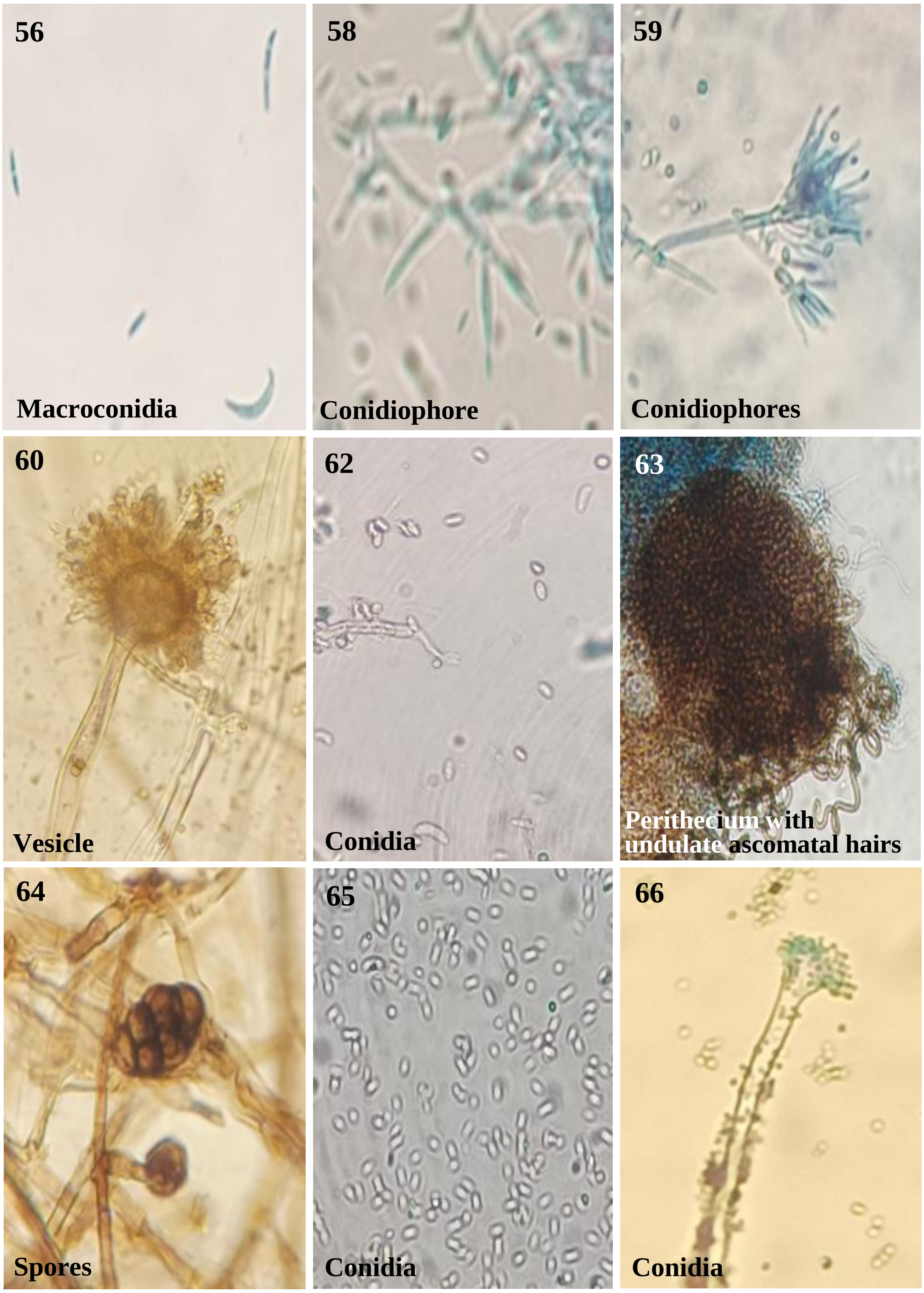
56
Macroconidia
58
Conidiophore
59
Conidiophores
60
Vesicle
62
Conidia
63
Perithecium with
undulate ascomatal hairs
64
Spores
65
Conidia
66
Conidia

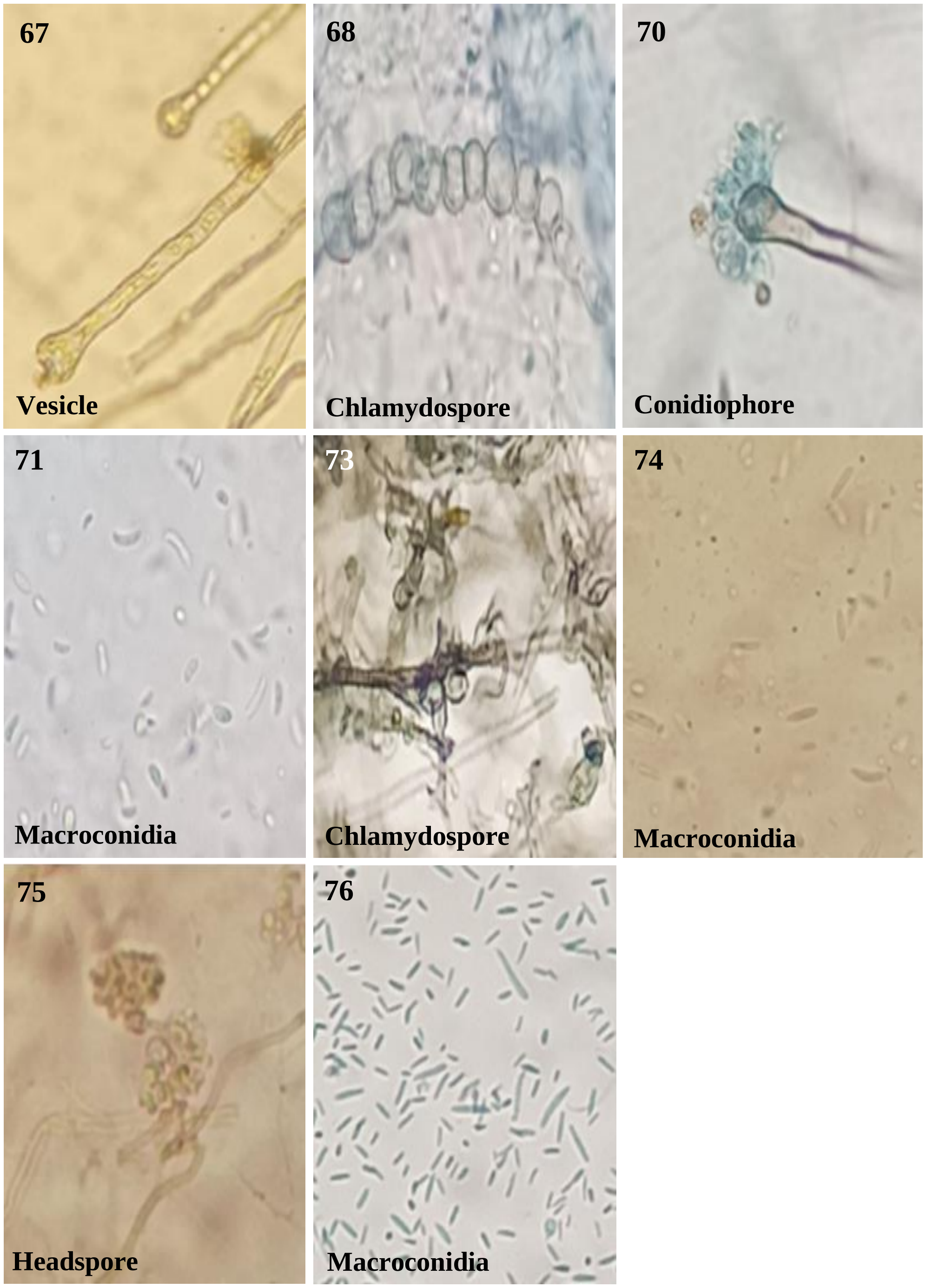

Figure S3. Photomicrographs of representative isolates of different morphotypes, isolated from *Ferula ovina*, *F. galbaniflua*, and *F. persica* tissues, considered for detailed description and morphological and molecular identification

Table S2. Accession numbers of the partial sequences of the fungal internal transcribed spacer (ITS) fragments (ITS1-5.8S-ITS2) obtained from representative isolates of different morphotypes, isolated from *Ferula ovina*, *F. galbaniflua*, and *F. persica* tissues, deposited in GenBank (NCBI)

Morphotype	Fungal genus	Accession number	Morphotype	Fungal genus	Accession number
1	*Aspergillus*	PP564394	40	*Aspergillus*	PP574213
2	*Zopfia*	PP574175	41	*Preussia*	PP574214
3	*Alternaria*	PP574176	42	*Fusarium*	PP574215
4	*Botrytis*	PP574177	43	*Cladosporium*	PP574216
5	*Albifimbria*	PP574178	44	*Aspergillus*	PP574217
6	*Bulbithecium*	PP574179	45	*Chaetomium*	PP574218
7	*Phoma*	PP574180	46	*Chaetomium*	PP574219
8	*Fusarium*	PP574181	47	*Chaetomium*	PP574220
9	*Cadophora*	PP574182	48	*Chaetomium*	PP574221
10	*Aspergillus*	PP574183	49	*Penicillium*	PP574222
11	*Fusarium*	PP574184	50	*Botrytis*	PP574223
12	*Aspergillus*	PP574185	51	*Fusarium*	PP574224
13	*Dactylonectria*	PP574186	52	*Zopfia*	PP574174
14	*Fusarium*	PP574187	53	*Acremonium*	PP574225
15	*Zopfia*	PP574188	54	*Penicillium*	PP574226
16	*Phoma*	PP574189	55	*Gibellulopsis*	PP574227
17	*Acremonium*	PP574190	56	*Fusarium*	PP574228
18	*Gliocladium*	PP574191	57	*Trichocladium*	PP574229
19	*Alternaria*	PP574192	58	*Fusarium*	PP574230
20	*Dothiorella*	PP574193	59	*Penicillium*	PP574231
21	*Parvothecium*	PP574194	60	*Aspergillus*	PP574232
22	*Acremonium*	PP574195	61	*Brunneochlamydosporium*	PP574233
23	*Talaromyces*	PP574196	62	*Fusarium*	PP574234
24	*Gliocladium*	PP574197	63	*Chaetomium*	PP574235
25	*Acremonium*	PP574198	64	*Alternaria*	PP574236
26	*Fusarium*	PP574199	65	*Fusarium*	PP564425
27	*Ganoderma*	PP574200	66	*Aspergillus*	PP574237
28	*Aspergillus*	PP574201	67	*Aspergillus*	PP574238
29	*Cadophora*	PP574202	68	*Acremonium*	PP574239
30	*Penicillium*	PP574203	69	*Dactylonectria*	PP574240
31	*Alternaria*	PP574204	70	*Aspergillus*	PP574241
32	*Cadophora*	PP574205	71	*Fusarium*	PP564428
33	*Cladosporium*	PP574206	72	*Acremonium*	PP574242
34	*Trichocladium*	PP574207	73	*Zopfia*	PP574247
35	*Cadophora*	PP574208	74	*Fusarium*	PP574243
36	*Acremonium*	PP574209	75	*Acremonium*	PP574244
37	*Gliomastix*	PP574210	76	*Fusarium*	PP574245
38	*Fusarium*	PP574211	77	*Fusarium*	PP574246
39	*Aspergillus*	PP574212			

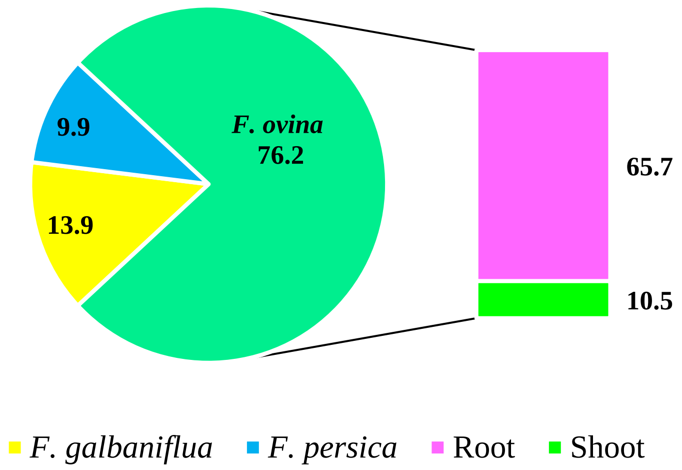

Figure S4. Relative abundance (%) of endophytic fungi isolated *Ferula ovina*, *F. galbaniflua* and *F. persica*

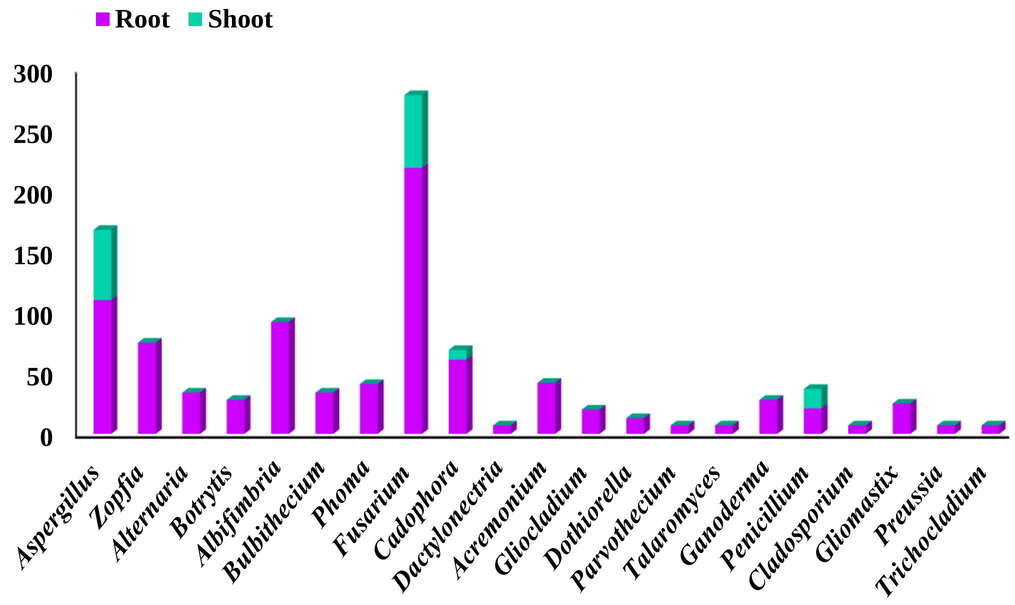

Figure S5. The genera number of endophytic fungi isolated from the shoots and roots of *Ferula ovina*

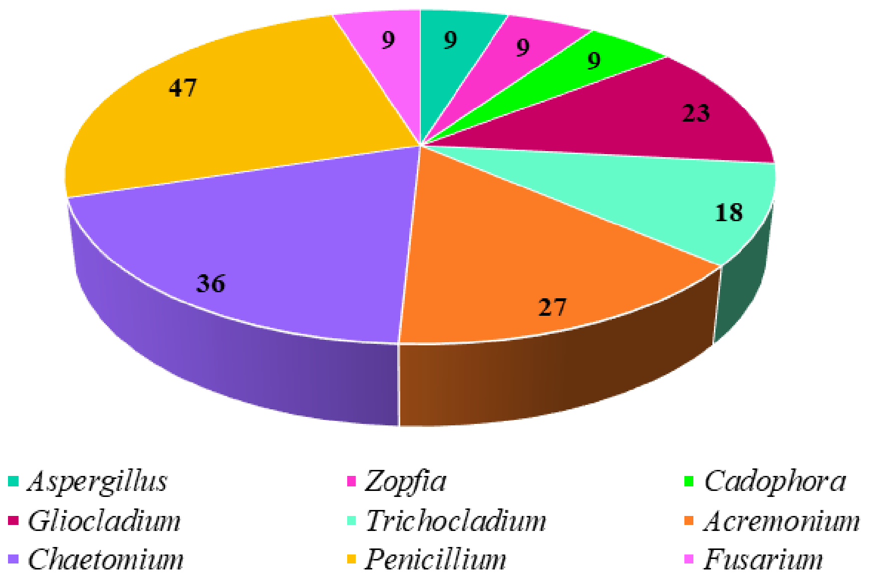

Figure S6. The genera number of endophytic fungi isolated from the roots of *Ferula galbaniflua*

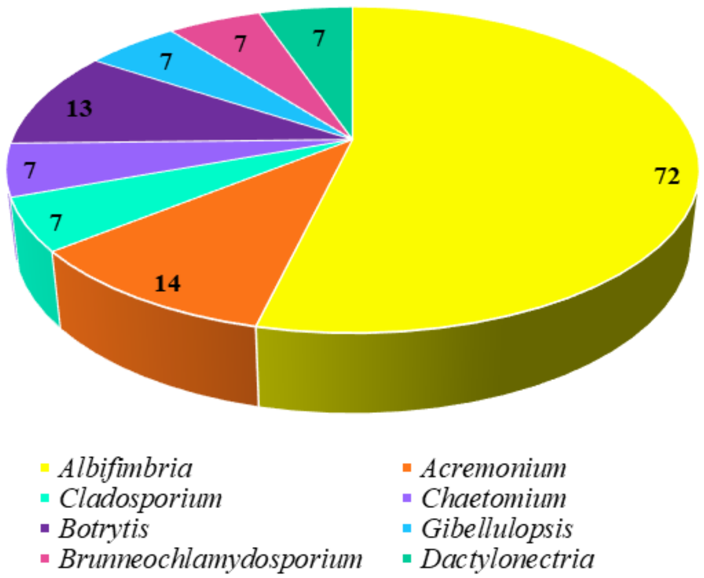

Figure S7. The genera number of endophytic fungi isolated from the roots of *Ferula persica*

Table S3. Number of fungal species isolated from *Ferula ovina*, *F. galbaniflua*, and *F. persica*

Variables	***F. ovina*** **roots**	***F. ovina*** **shoots**	***F. ovina*** **(roots and shoots)**	***F. galbaniflua*** **roots**	***F. persica*** **roots**
**Total no. of species (S)**	57	10	60	17	9
**Total no. of individual (N)**	885	142	1027	187	134
**Natural log of species (ln S)**	4.04	2.30	4.09	2.83	2.20
**Natural log of individual (ln N)**	6.78	4.956	6.93	5.23	4.898

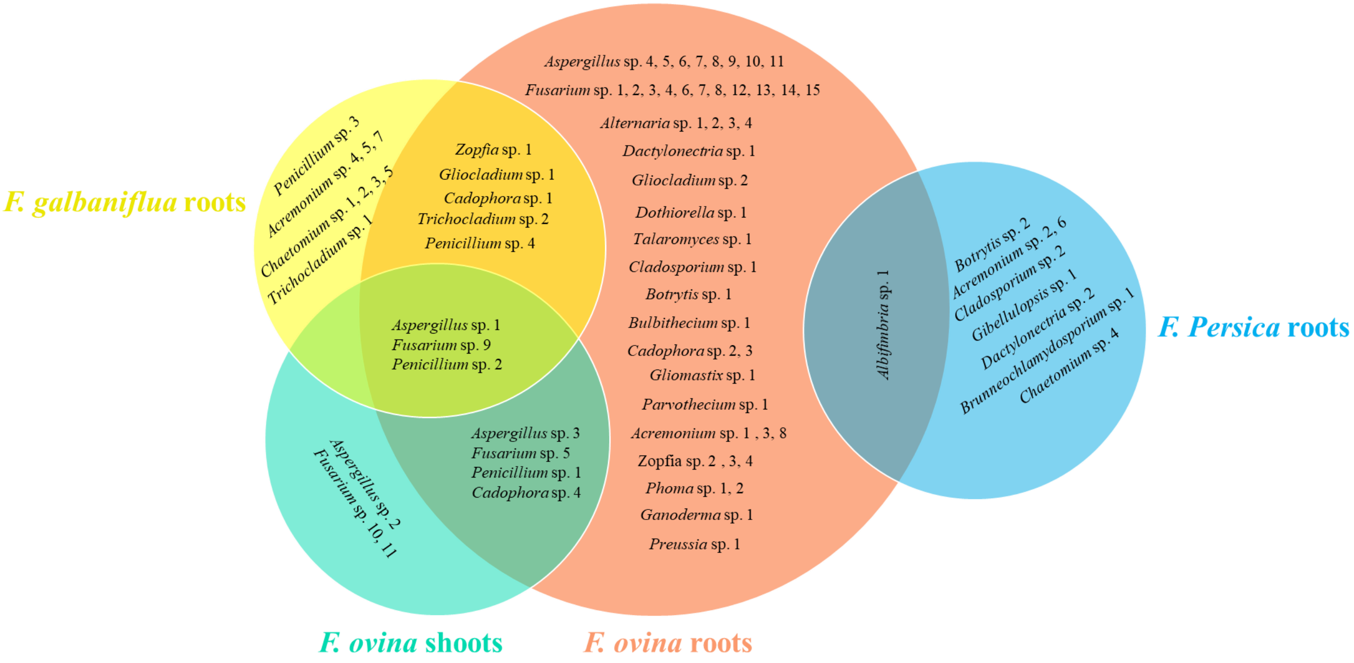

Figure S8. Venn diagram of endemic and common endophytic fungal species isolated from *Ferula ovina* roots and shoots, *F. galbaniflua* roots, *F. persica* roots

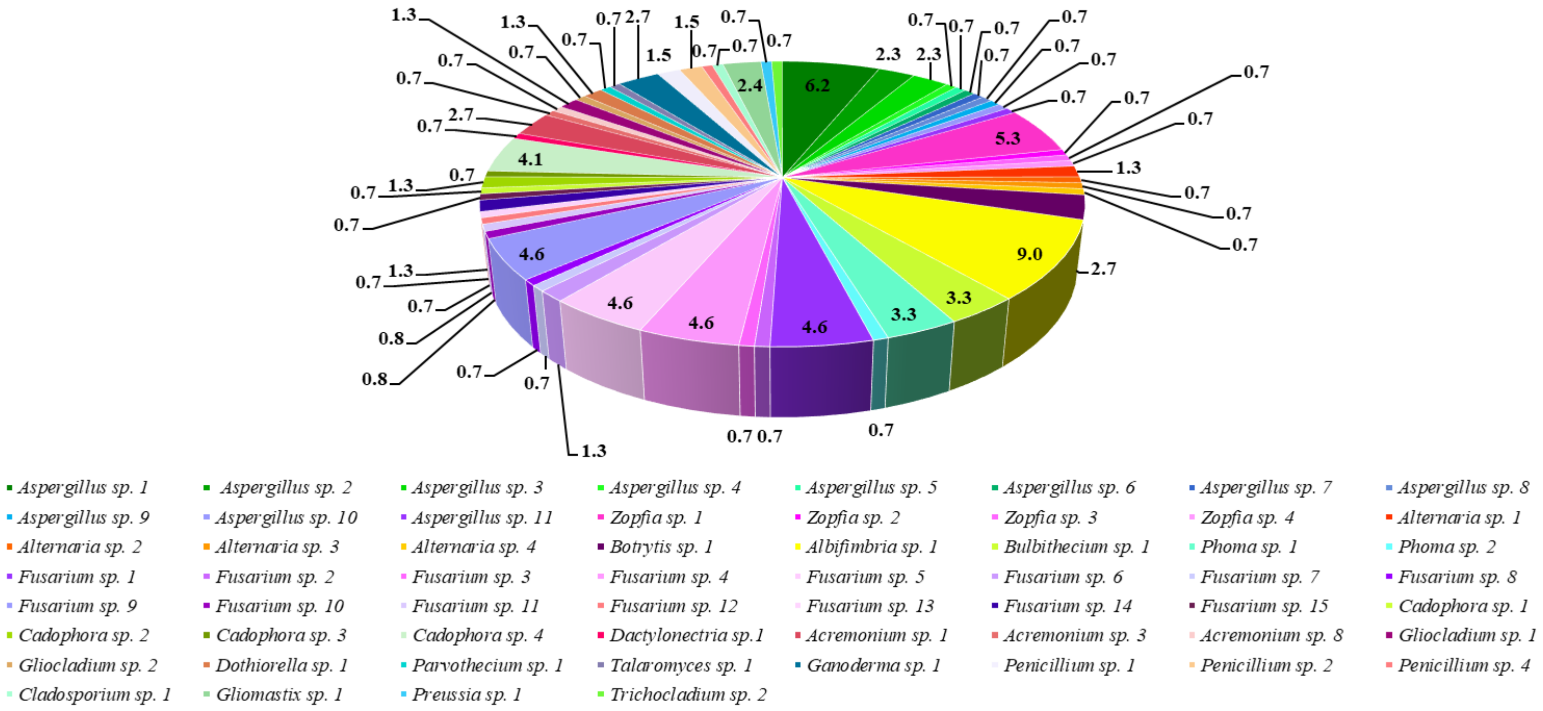

Figure S9. Relative frequency of endophytic fungi isolated from *Ferula ovina* roots and shoots

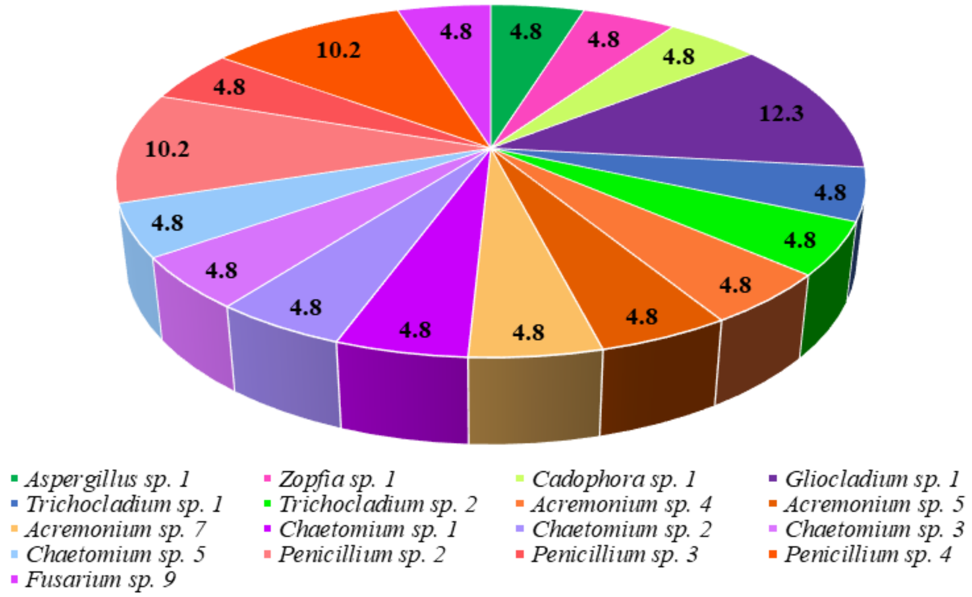

Figure S10. Relative frequency of endophytic fungi isolated from *Ferula galbaniflua* roots

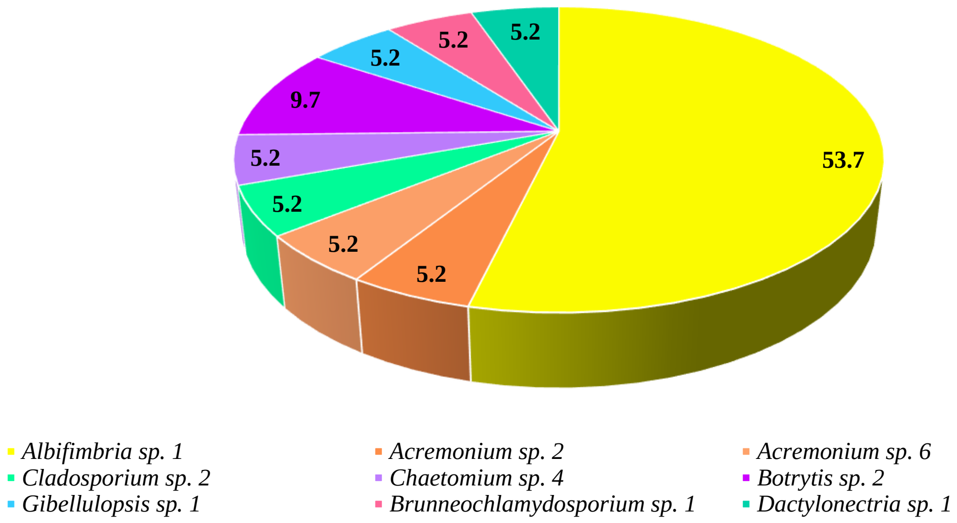

Figure S11. Relative frequency of endophytic fungi isolated from *Ferula persica* roots

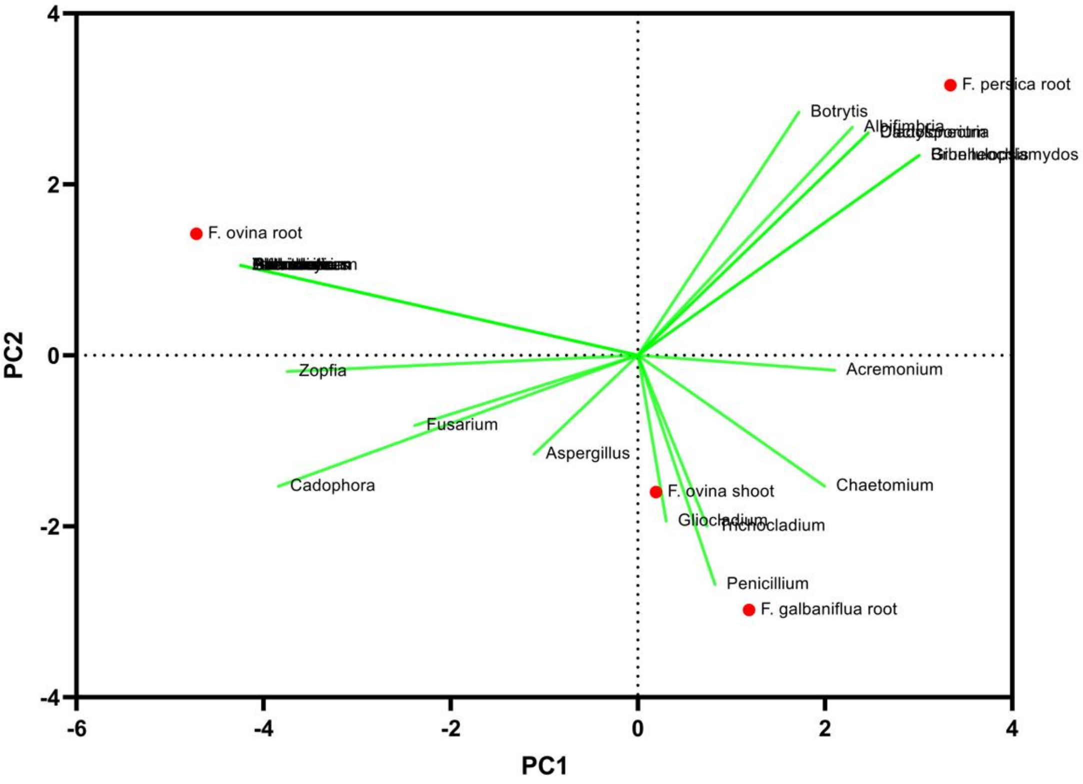

Figure S12. Principal component analysis: Position of *Ferula ovina* roots and shoots, *F. galbaniflua* roots, and *F. persica* roots based on the relative abundance of endophytic fungal genera isolated from them.
